# What works to reduce sedentary behavior in the office, and could these intervention components transfer to the home working environment?: A rapid review and transferability appraisal

**DOI:** 10.3389/fspor.2022.954639

**Published:** 2022-07-29

**Authors:** Sarah Morton, Claire Fitzsimons, Ruth Jepson, David H. Saunders, Divya Sivaramakrishnan, Ailsa Niven

**Affiliations:** ^1^Physical Activity for Health Research Centre, University of Edinburgh, Edinburgh, United Kingdom; ^2^Scottish Collaboration of Public Health Research & Policy, University of Edinburgh, Edinburgh, United Kingdom

**Keywords:** sitting, occupational, home working, flexible working, behavior change

## Abstract

**Background:**

Working patterns have changed dramatically due to COVID-19, with many workers now spending at least a portion of their working week at home. The office environment was already associated with high levels of sedentary behavior, and there is emerging evidence that working at home further elevates these levels. The aim of this rapid review (PROSPERO CRD42021278539) was to build on existing evidence to identify what works to reduce sedentary behavior in an office environment, and consider whether these could be transferable to support those working at home.

**Methods:**

The results of a systematic search of databases CENTRAL, MEDLINE, Embase, PsycInfo, CINHAL, and SportDiscus from 10 August 2017 to 6 September 2021 were added to the references included in a 2018 Cochrane review of office based sedentary interventions. These references were screened and controlled peer-reviewed English language studies demonstrating a beneficial direction of effect for office-based interventions on sedentary behavior outcomes in healthy adults were included. For each study, two of five authors screened the title and abstract, the full-texts, undertook data extraction, and assessed risk of bias on the included studies. Informed by the Behavior Change Wheel, the most commonly used intervention functions and behavior change techniques were identified from the extracted data. Finally, a sample of common intervention strategies were evaluated by the researchers and stakeholders for potential transferability to the working at home environment.

**Results:**

Twenty-two studies including 29 interventions showing a beneficial direction of effect on sedentary outcomes were included. The most commonly used intervention functions were training (*n* = 21), environmental restructuring (*n* = 21), education (*n* = 15), and enablement (*n* = 15). Within these the commonly used behavior change techniques were instructions on how to perform the behavior (*n* = 21), adding objects to the environment (*n* = 20), and restructuring the physical environment (*n* = 19). Those strategies with the most promise for transferring to the home environment included education materials, use of role models, incentives, and prompts.

**Conclusions:**

This review has characterized interventions that show a beneficial direction of effect to reduce office sedentary behavior, and identified promising strategies to support workers in the home environment as the world adapts to a new working landscape.

**Systematic Review Registration:** https://www.crd.york.ac.uk/prospero/display_record.php?ID=CRD42021278539, identifier CRD42021278539.

## Introduction

Sedentary behavior is defined as any waking behavior characterized by an energy expenditure ≤1.5 METs while in a sitting, reclining or lying posture (Tremblay et al., 2017). In 2020, the World Health Organization (WHO) recommended that all adults should limit the amount of time they spend being sedentary (World Health Organization, 2020). This recommendation was based on evidence that higher levels of sedentary behavior increase the risk of adverse physical health outcomes including all-cause, cardiovascular disease and cancer mortality, and the incidence of cardiovascular disease, cancer and type 2 diabetes (Dempsey et al., 2020). Importantly, these physical health risks of being highly sedentary are attenuated only with relatively high levels of physical activity (Dempsey et al., 2020). Additional evidence indicates that higher levels of sedentary behavior are also associated with adverse mental health outcomes (Huang et al., 2020; Biddle et al., 2021).

The workplace is a setting associated with high levels of sedentary behavior, with evidence that office-based employees can spend up to 82% of their working day sitting (Parry and Straker, 2013; Hadgraft et al., 2016; Maes et al., 2020; Rosenkranz et al., 2020); equivalent of up to 438 min/day (Parry and Straker, 2013). Consequently, there has been a growth in intervention research designed to support employees to reduce their sitting whilst at work. A recent 2018 Cochrane review of workplace interventions for reducing sitting at work (*n* = 34 studies; *n* = 3,397 participants) concluded that there was some evidence for the short-term (i.e., <12 month) benefits of sit-stand desks on reducing time spent sitting (Shrestha et al., 2018). There was insufficient evidence to draw conclusions on the effectiveness of other intervention strategies. The authors highlighted the low quality of the studies and need for further research. However, in the 4 years since this review, there have been a number of other high quality intervention studies, with more beneficial outcomes (e.g., Healy et al., 2016; Edwardson et al., 2018).

Although the findings from these studies are valuable, due to COVID-19 there has been a seismic shift in working patterns in many sectors that requires consideration. In many countries, lockdown restrictions required employees, where possible, to work from home; and there is indication that these restrictions have resulted in permanent changes in working patterns. For example, in the UK it is anticipated that many employees will spend at least a portion of the working week in the home environment through a hybrid home and office working approach, as we adapt to a “new normal” working landscape (British Council for Offices, 2020; BBC News, 2022).

Whilst there are benefits to working at home for some employees (e.g., reduced commuting time and cost, enhanced work-life balance; Vyas and Butakhieo, 2020), there is initial evidence that suggests this shift to working at home has increased sedentary time. For example, compared with not working at home, working at home during COVID-19 was associated with between ~31 (McDowell et al., 2020) and 110 min (or 24% of working time) (Fukushima et al., 2021) more sitting time per working day. Additionally, in a series of studies in a single workplace evaluating the impact of introducing flexible working (i.e., being able to work remotely away from the office), Olsen and colleagues reported an increase in actual and perceived workplace sitting time when workers were not in the office (Olsen et al., 2018a,b).

Given that working at home appears to have exacerbated already high levels of sitting time exhibited by office-based employees, there is an urgent need to identify potential intervention strategies to support workers as they adapt to this new work setting. The Behavior Change Wheel (BCW) (Michie et al., 2011, 2014) provides a useful framework for intervention development, which has been successfully used in office-based sedentary research (Munir et al., 2018; Ojo et al., 2019). In short, the BCW involves three stages of intervention development. In the first stage, developers specify the target behavior and identify the sources that influence the behavior. The COM-B model is used to guide identification of the role **C**apability, **O**pportunity and **M**otivation as influential sources on **B**ehavior, and what needs to change. In stage 2, what needs to change is mapped to appropriate intervention functions (e.g., education) and policy categories (e.g., communication/marketing). Finally, in stage 3, developers specify the content of the intervention by identifying which behavior change techniques (BCTs) best serve the intervention functions, and how they should be delivered. A BCT is defined as “an active component of an intervention designed to change behavior” (p. 145). Michie et al. have presented a taxonomy of 93 BCTS, including for example, goal setting and social support (Michie et al., 2013).

In addition to using the BCW to design interventions, sedentary behavior researchers have also used the BCW as a framework to retrospectively examine pre-existing interventions and to determine which BCW intervention functions and BCTs were included, and which were most effective (Gardner et al., 2016; Dunn et al., 2018; Curran et al., 2021). For example, Gardner et al. (2016) reported that workplace interventions incorporating environmental restructuring and education were most promising, and that a range of BCTs may be useful. Given the recent growth of high-quality office-based sedentary behavior intervention research, there is considerable value in identifying which BCW intervention functions and BCTs are associated with effectiveness in this setting. This information could provide a useful and efficient starting point for intervention development in the new home working environment. Indeed, several intervention development frameworks (e.g., Michie et al., 2011; Skivington et al., 2021) recognize that interventions from existing contexts may be adaptable to new contexts.

However, it is acknowledged that the home working environment differs from the office environment, and that those interventions effective in an office setting may not be directly transferable to the home. For example, at home the physical space available to move around, and financial and social resources available to support behavior change may be more limited. Therefore, it is important to also evaluate the potential transferability of effective BCW intervention functions and BCTs in the office to the working at home environment. Within the BCW framework, Michie et al. (Michie et al., 2014; West and Michie, 2022) outline the APEASE criteria that may be applied during intervention development to evaluate the application of similar interventions to a different context. Using the criteria, intervention developers assess the **A**ffordability, **P**racticability, **E**ffectiveness, **A**cceptability, **S**ide-effects, and **E**quity of proposed interventions to assess their potential for transferability to a different context (e.g., Jenkins et al., 2018).

In summary, the revolutionary changes in working practices initiated by COVID-19 have led to considerable growth in working at home. However, this environment appears to be a high risk setting for unhealthy sedentary behavior, and there is a need to support workers to reduce prolonged sitting when working at home. Given the lack of intervention research in this context, there is benefit and efficiency in drawing from the growing evidence base on office-based sedentary interventions. Particularly, findings from high quality studies of intervention strategies that show a beneficial effect will enable identification of what types of interventions work in the office environment, and through appraisal, consideration of the transferability to the home working environment.

Therefore, the overall aim of this rapid review was to identify the types of interventions that have been beneficial in reducing sedentary behavior in healthy adult workers in an office environment, and to appraise the opportunity for transferability to the home working environment. There were three objectives:

1. To identify interventions with a beneficial direction of effect in reducing sedentary behavior in office-based settings.
2. To use the BCW to identify and classify the most commonly used intervention functions and BCTs of interventions with a beneficial direction of effect.
3. In consultation with expert stakeholders, to appraise the potential transferability of components of office-based interventions with a beneficial direction of effect to the home working environment using the APEASE criteria.

## Methods

This review was pre-registered to the PROSPERO database (reference number CRD42021278539). In the absence of specific guidelines for reporting of rapid reviews, this study was reported in accordance with PRISMA 2020 guidelines (Page et al., 2021) for reporting systematic reviews (see Supplementary File 1 for PRISMA checklist).

### Search methods

Consistent with the Cochrane Rapid Reviews Methods Group guidance (Garritty et al., 2021) a stepwise approach was adopted, and an existing relevant Cochrane systematic review was used as our starting point (Shrestha et al., 2018). We incorporated the studies included in the 2018 Cochrane review into our title and abstract screening stage. We then searched the Cochrane Central Register of Controlled Trials (CENTRAL), MEDLINE, Embase, PsycInfo, CINHAL, and SportDiscus from the last search date of the Cochrane review, which was 10 August 2017, to 06 September 2021. Table 1 shows the search strategy adopted.

**Table 1:** Rapid review search strategy by database.

**CENTRAL**
#1 work* #2 sedentary #3 sitting #4 #2 or #3 #5 office #6 inactiv* #7 #5 and #6 #8 #4 or #7 #9 #1 and #8 #10 #9 AND trials LIMITS: August 2017 – Sept 2021; trials
**MEDLINE**
#1 (work[tw] OR works*[tw] OR work'*[tw] OR worka*[tw] OR worke*[tw] OR workg*[tw] OR worki*[tw] OR workl*[tw] OR workp*[tw] OR occupation*[tw] OR employe*[tw]) #2 (effect*[tw] OR control[tw] OR controls*[tw] OR controla*[tw] OR controle*[tw] OR controli*[tw] OR controll*[tw] OR evaluat*[tw] OR intervention*[tw] OR program*[tw] OR compare*[tw]) #3 (sedentary OR sitting) OR seated posture OR chair[tiab] OR desk[tiab] OR (office AND inactiv*) #4 (animals [mh] NOT humans [mh]) #5 #1 AND #2 AND #3 NOT #4 LIMITERS: 2017-current; humans; English language
**Embase**
#1 sedentary—changed to sedentar* #2 'sitting'/de—changed to sit* #3 'seated posture' #4 seated NEAR/1 posture (term rejected) – changed to seated adj3 posture #5 chair:ab,ti OR desk:ab,ti #6 chair:ab,ti #7 desk:ab,ti #8 office AND inactiv* #9 #1 OR #2 OR #4 OR #6 OR #7 OR #8 #10 'work'/de OR work #11 work* #12 'occupation'/de OR occupation—changed to occupation* #13 employe* #14 #10 OR #12 OR #13 #15 effect #16 control #17 evaluat* #18 intervention* #19 program #20 compare #21 #15 OR #16 OR #17 OR #18 OR #19 OR #20 #22 #9 AND #14 AND #21 #23 #22 AND [embase]/lim #24 #23 AND [humans]/lim AND [embase]/lim
LIMITERS: 2017-current; humans; English language; Embase: journal; article; 18-64 years
**CINAHL**
S10 S1 AND S2 AND S9 Limiters - Exclude MEDLINE records Search modes - Boolean/Phrase S9 S3 OR S4 OR S5 OR S6 OR S7 OR S8 S8 (office AND inactive*) or TX (office AND inactive*) or MW (office AND inactive*) S7 Desk or TX desk or MW desk S6 Sedentary or TX sedentary or MW sedentary S5 Seated posture or TX seated posture or MW seated posture S4 Sitting or TX sitting or MW sitting S3 Chair or TX chair or MW chair S2 TX randomized controlled trial or TX controlled clinical trial or AB placebo or TX clinical trials or AB randomly or TI trial or TX intervent* or control* or evaluation* or program* S1 work* OR (offic* OR busines*) OR occupat* LIMITERS: 2017-current; humans; English language; 19-44 and 45-64 years
**PsycINFO**
S25 S13 AND S17 AND S24 S24 S18 OR S19 OR S20 OR S21 OR S22 OR S23 S23 compare S22 program S21 intervention* S20 evaluat*
S19 control
S18 effect
S17 S14 OR S15 OR S16
S16 employe*
S15 occupation
S14 work
S13 S1 OR S2 OR S4 OR S8 OR S11 OR S12
S12 office AND inactive*
S11 S9 OR S10
S10 ab(desk)
S9 ti(desk)
S8 S6 OR S7
S7 ti(chair)
S6 ab(chair)
S5 ab(chair) OR ti(chair)
S4 seated NEAR/1 posture – changed to seated adj3 posture
S3 seated posture
S2 sitting
S1 sedentary
LIMITERS: 2017 – current; English; humans
**SportDiscus**
S10 S1 AND S2 AND S9
S9 S3 OR S4 OR S5 OR S6 OR S7 OR S8
S8 (office AND inactive*) or TX (office AND inactive*) or MW (office AND inactive*)
S7 Desk or TX desk or MW desk
S6 Sedentary or TX sedentary or MW sedentary S5 Seated posture or TX seated posture or MW seated posture S4 Sitting or TX sitting or MW sitting S3 Chair or TX chair or MW chair S2 TX randomized controlled trial or TX controlled clinical trial or AB placebo or TX clinical trials or AB randomly or TI trial or TX intervent* or control* or evaluation* or program* S1 work* OR (offic* OR busines*) OR occupat* LIMITERS: 2017-2021; ENGISH, ACADEMIC JOURNALS

### Eligibility criteria

The inclusion criteria for eligible studies were peer reviewed publications in English language that included: (a) healthy (i.e., not recruited to a study focusing on a specific health-related condition, such as back-pain or obesity) adults aged 18 and over; (b) interventions to reduce occupational sitting in office-based settings; (c) a true control arm comparison (i.e., control condition for cross-over design or control group for between subject design; (d) an outcome assessing sedentary behavior during the normal working day reported using either self-reported measures (e.g., activity log, questionnaire) or device measured (e.g., accelerometer) or both, including changes in at least one of: time spent sitting, time spent standing, posture, and number of sitting breaks; (e) a randomized controlled trial (RCTs), cross-over RCT, or cluster RCT design. We only included interventions that showed a beneficial direction of effect for at least one sedentary behavior outcome in comparison to the control at any post-baseline time point, whether or not statistically significant. This criterion was adopted at the full-text stage of screening to be inclusive of interventions with potential, and due to the known shortcomings of relying on statistical significance to make judgements of effectiveness, especially when there are data from low numbers of participants (Wasserstein et al., 2019).

### Screening and identification of interventions with a beneficial direction of effect

Covidence review software was used to facilitate study identification. References were imported (including publications associated with the 34 studies from the 2018 Cochrane review), and duplicates identified and removed prior to commencing screening. All five reviewers (AN, SM, CF, DS, and DSi) were involved at each step. Each title and abstract was independently screened by two of the five reviewers. Where titles and abstracts met the eligibility criteria, full texts were located. Screening of a sample of five full text articles was conducted by all team members to calibrate and test the review form. Subsequently, all full-text papers were independently screened by two of the five reviewers, and any disagreements were resolved through discussion within the research team.

### Data extraction of study characteristics and assessment of risk of bias

For each included study the data were extracted by one reviewer (SM) to a bespoke excel spreadsheet. The extracted data were subsequently reviewed and checked by one of the four other reviewers (AN, CF, DS, and DSi). Extracted data included general study information, information on study participants (including those included in the analysis of the intervention with a beneficial direction of effect), sedentary behavior measurement instrument, intervention characteristics, and changes in occupational sedentary behavior at assessed time-points post-baseline.

Risk of Bias (RoB) assessment was completed in Covidence using the Cochrane RoB tool and guidance (Higgins et al., 2011). Again, one reviewer (SM) independently conducted RoB for all included studies, and then allocated studies equally across the rest of the team (AN, CF, DS, DSi), each of whom completed the same RoB process independently, then met with SM to discuss and agree final assessments. Included studies were assessed according to (i) sequence generation; (ii) allocation concealment; (iii) blinding of outcome assessments; (iv) incomplete outcome data; (v) selective reporting; (vi) validity of outcome reporting; (vii) baseline comparability/imbalance for age, gender, and occupation of study groups. Each potential source of bias was graded as either high risk (i.e., if there was sufficient detail to demonstrate procedures leading to high risk of bias), unsure (i.e., if there was insufficient detail to make a decision), or low risk (i.e., if there was sufficient detail that high quality procedures had been followed).

### Using the BCW to identify and classify the most commonly used intervention functions and BCTs

In order to systematically classify the content of each effective intervention, individual components were extracted and mapped to the BCW intervention functions, and this was undertaken at study level. The intervention functions were defined as articulated by Michie et al. (2014) and included education, persuasion, incentivisation, coercion, training, restrictions, environmental restructuring, modeling, and enablement. We then identified the individual BCTs (Michie et al., 2013) within each BCW intervention component, and the delivery mechanism. These steps were undertaken by one reviewer, checked by a second, and discussed until consensus was reached. Three reviewers (AN, CF, and SM) had undertaken BCT Taxonomy training (bct-taxonomy.com), and at least one of these was involved in each step. Given the potential presence of up to 93 BCTs, in order to adopt a parsimonious approach we focused primarily on those BCTs previously identified as being most commonly used to address the different intervention functions (Michie et al., 2011, 2014).

### Using the APEASE criteria to appraise the potential transferability of intervention components

The APEASE criteria were applied by one reviewer and checked by a second for each study to evaluate the transferability of interventions from an office to a home working environment. For each study, we rated the transferability of the included BCTs as transferable, possibly transferable, or not transferable across each of the APEASE criteria.

Following this step, we engaged with seven expert stakeholders to invite their feedback on the potential transferability of the identified intervention functions. These stakeholders had already been invited to be part of a larger research project focusing on developing interventions to reduce sedentary behavior when working at home. These experienced stakeholders were included because they had a remit for workplace health in Scotland, were likely to be involved in the delivery of any resultant intervention (Skivington et al., 2021), and were willing to be involved. Our stakeholders included a senior development officer, and a development officer in workplace health from a Scottish charity that promotes walking for health, employees of The Scottish Government [who had a remit for occupational health (*n* = 2) and for strategy (*n* = 1)], a representative from the health promotion service in Public Health Scotland, and an active travel project officer from a UK charity with a remit for walking and cycling. Initially, we hosted an online meeting with the stakeholders to provide an overview of the context and guidance on applying the APEASE framework. The stakeholders then completed an online questionnaire that listed all of the BCW intervention functions used in the included studies, and associated examples of how the intervention had been delivered in practice. Experts were asked to provide a score of 1, 2, or 3 for each APEASE criteria to indicate transferability from an office environment to a home working environment. A score of 1 indicated transferable; 2 indicated possibly transferable; 3 indicated not transferable. The majority score for each example was identified. Following research team discussion, SM then completed scoring on behalf of the research team. This was checked and discussed with another reviewer (DSi) until consensus was reached. The criterion of effectiveness was excluded since it is based on an evaluation of the intervention in a specific setting, and these interventions have not yet been tested in the home working environment. For ease of presentation and interpretation, the 1–3 scoring was translated as follows: 1 to “+” to represent transferable; 2 to “?” for potentially transferable, and 3 was translated to “–” to represent not transferable.

## Results

### Inclusion of studies

**Figure 1:**
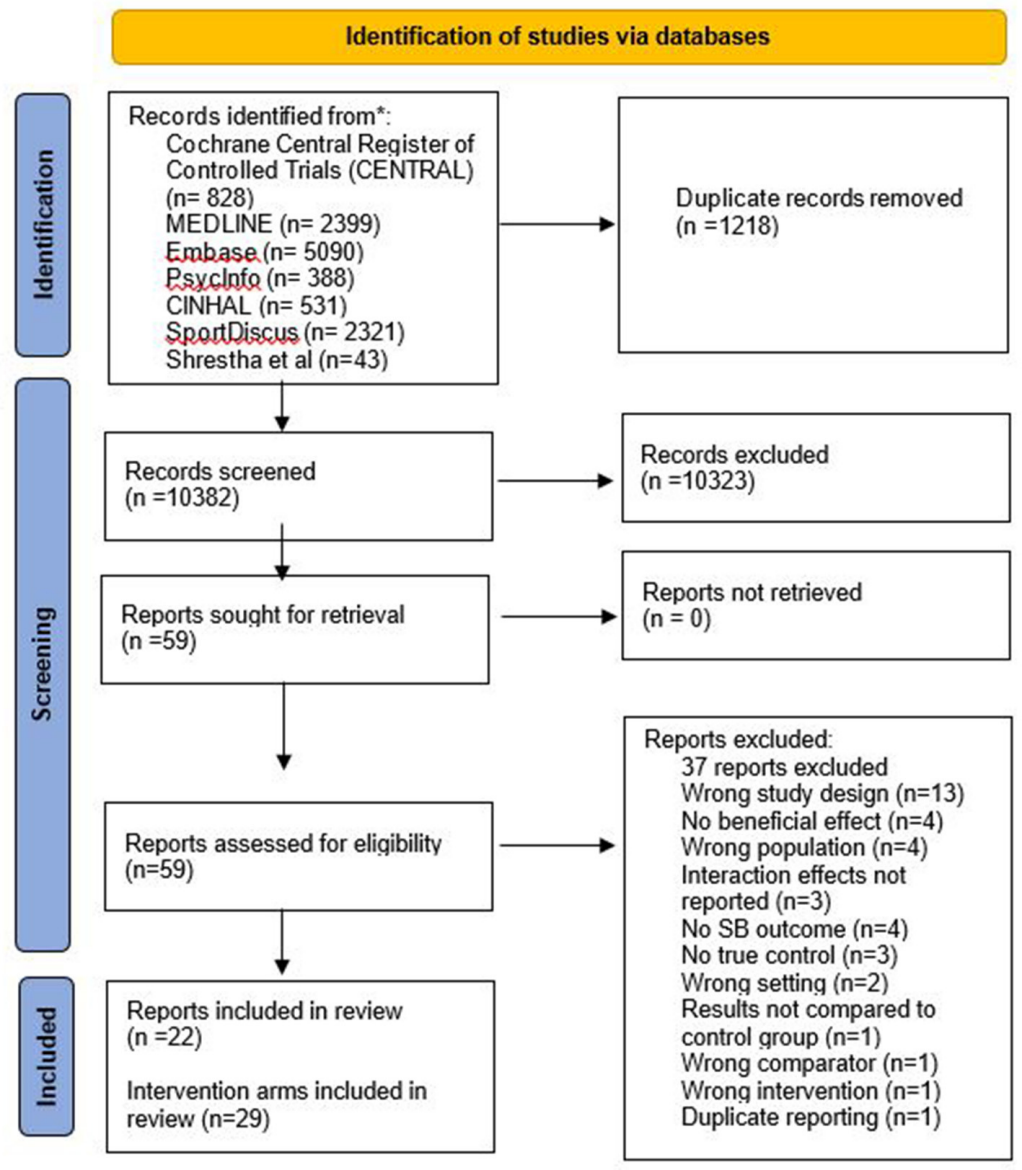
PRISMA flow diagram obtained from Covidence showing the study selection process.

The PRISMA study flow diagram (Page et al., 2021; Figure 1) details the process of identifying the included studies. A total of 11,557 references were identified during the initial search of six databases, and we also included 43 papers (from 34 studies) included in the 2018 Cochrane review of office-based interventions (Shrestha et al., 2018). We imported 11,600 titles to Covidence for screening. After duplicates were removed, 10,382 titles and abstracts were screened, and 59 reports retrieved for full text screening. Of these, we excluded 37 papers and included 22 in this review. Five studies had more than one intervention arm demonstrating a beneficial direction of effect, consequently 29 interventions arms were included in the review. Of the 34 studies included in the Cochrane review, 11 were included in our review (Chau et al., 2014; Coffeng et al., 2014; Dutta et al., 2014; Neuhaus et al., 2014; Graves et al., 2015; Puig-Ribera et al., 2015; De Cocker et al., 2016; Healy et al., 2016; Tobin et al., 2016; Danquah et al., 2017; Li et al., 2017).

### Characteristics of studies demonstrating a beneficial direction of effect on sedentary behavior

#### Participants

Table 2 describes the characteristics of the 22 included studies. A total of 1,577 participants were included across the 29 intervention arms, with sample sizes at baseline ranging from 6 (Li et al., 2017) to 196 (Blake et al., 2019) participants, and with an average of 49 (±46) participants in each intervention arm. All participants worked in an office-based role (e.g., administrative, financial services, managerial, customer services) from public and private sectors including universities, government, an environmental agency, a health promotion unit, construction services, an energy company, and a national health service. The studies were undertaken in 13 countries including China, Australia, Netherlands, Denmark, Greenland, Belgium, South Africa, USA, Canada, England, India, Spain, or New Zealand. Sixteen studies reported the gender composition of the sample, and in 14 of these studies there was a higher proportion of female participants. All participants were of working age 18–65 years old, and the mean age of the participants was 40.86 (±3.81) years. All participants were healthy and spent most of the working day in a seated posture, for example at a desk, computer, or workstation.

**Table 2:** Characteristics of included studies showing a beneficial direction of effect.

**References**	**Participants of intervention group**				**Measurement tool**	**Beneficial direction of effect in**			
						**occupational sedentary behavior**			
	**Sector**	**Country**	**Age**	**Number and gender**		**Number of breaks**	**Sitting**	**Posture change**	**Standing**
Blake et al. (2019)	Private sector IT	China	X	Baseline: *n* = 196, 49.5% F 27% loss to follow-up *n* = 143 % F not reported	Self-report weekday sitting hours	Not reported	✓	Not reported	Not reported
Carter et al. (2020)	University	England	42.5 (± 10.0)	Baseline: *N* = 14 57.1% F *N* = 6 intervention completed post assessment *N* = 9 crossover intervention completed post assessment; % F not reported	ActivPAL	Not reported	✓	✓	✓
Chau et al. (2014)	Non-government health agency	Australia	38 (±11)	Baseline: *N* = 42 86% F Pre-INT 1 *n* = 33 Pre-INT 2 *n* = 39 Post-INT *n* = 38	ActivPAL OSPAQ [1] WSQ [2]	Not reported	✓	Not reported	✓
Coffeng et al. (2014)	Financial services	Netherlands			Unvalidated self-report: estimate the total amount of minutes spend at				
a) Social and physical environmental intervention			38 (±10.5)	Baseline: *N* = 92 44.6% F *N* = 63 at 6 mths *N* = 63 at 12 mths	work on computer use, meetings and other sedentary activities during a usual working day	Not reported	✓	Not reported	Not reported
b) Social environment intervention			43.6 (±10.3)	Baseline: *N* = 118 38.1% F *N* = 100 at 6 mths *N* = 94 at 12 mths		Not reported	✓	Not reported	Not reported
c) Physical environmental intervention			42.2 (±10.5)	Baseline: *N* = 96 37.5% F *N* = 76 at 6 mths *N* = 76 at 12 mths		Not reported	✓	Not reported	Not reported
Danquah et al. (2017)	Municipalities and private workplaces	Denmark and Greenland	46 (±10)	Baseline: *N* = 173 61% F	ActiGraph GT3X+ accelerometer (+log) – workdays (work time and leisure time)	Not reported	✓	✓	✓
De Cocker et al. (2016)	University and environmental agency	Belgium			WSQ [2], ActivPAL, day log				
a) Tailored intervention			40.5 (±8.6)	Baseline: *N* = 78 67.9% F *N* = 43 at 1 mth *N* = 36/38 at 3 mths		✓	✓	Not reported	✓
b) Generic intervention			40.7 (±9.7)	Baseline: *N* = 84 67.9% F *N* = 75/41 at 1 mth *N* = 67/42 at 3 mths		✓	✓	Not reported	✓
Dunning et al. (2018)	University and CBD	South Africa	27.9 (±5.4)	Baseline: *n* = 11 64%F Follow up: *n* = 7	ActivPAL, ActiGraph GT3X+	Not reported	✓	Not reported	✓
Dutta et al. (2014)	Not reported	USA	40.4	*N* = 28 67.8% F	Modular Signal Recorder (MSR) accelerometer; OSPAQ [1]	Not reported	✓	Not reported	✓
Edwardson et al. (2018)	National Health Service Trust	England	41.7 (±11.0)	Baseline: *N* = 77 *N* = 62 at 12 mths % F not reported (for final analysis)	ActivPAL micro	Not reported	✓	Not reported	✓
Graves et al. (2015)	University	England	38.8 (± 9.8)	Baseline: *N* = 26 89% F (23) *N* = 26 at 4 wks *N* = 25 at 8 wks	Ecological momentary assessment (EMA) diaries	Not reported	✓	Not reported	✓
Healy et al. (2016)	Government organization	Australia	44.6 (±9.1)	Baseline: *N* = 135 65.4% F *N* = 123 at 3 mths *N* = 100 at 12 mths	ActivPAL3	Not reported	✓	Not reported	✓
Li et al. (2017)	Health Promotion Unit	Australia			ActivPAL, OSPAQ				
a) Group 2: 40 min sitting/20 min standing			46 (4)	Baseline and follow up: *N* = 6 83% F		Not reported	✓	Not reported	✓
b) Group 3: 30 min sitting/30 min standing			40 (13)	Baseline and follow up: *N* = 5 60% F		Not reported	✓	Not reported	✓
c) Group 4: 20 min sitting/40 min standing			41 (14)	Baseline and follow up: *N* = 6 100% F		Not reported	✓	Not reported	✓
Lithopoulos et al. (2020)	Local workplaces	Canada			Adapted version of sitting portion of IPAQ [3]—self reported	Not reported		Not reported	Not reported
a) Affective			41.87 (±10.35)	*N* = 28 42.9% F			✓		
b) Instrumental			42.42 (±11.78)	*N* = 43 72.1% F			✓		
Mantzari et al. (2019)	Genomics company and an NHS Foundation Trust consisting of two hospitals	England	Not reported	*N* = 9 in intervention arm at (Phase 3) (and full data) % F not reported	ActivPAL	Not reported	✓	✓	✓
Maylor et al. (2018)	National property, residential, construction, and services group organization	England	43.0 (39.4–46.7)	For workplace sitting and activity outcomes, Baseline *N* = 46 54% F 8-week *N* = 38	ActivPAL	Not reported	✓	✓	✓
Neuhaus et al. (2014)	University	Australia			ActivPAL				
a) Multi-component group			37.3 (±10.7)	Baseline: *N* = 16 100% F *N* = 12 at 3 mths		Not reported	✓	✓	✓
b) Workstations only group			43.0 (±10.2)	*N* = 14 78.6% F *N* = 13 at 3 mths		Not reported	✓	✓	✓
Patel et al. (2021)	University	India	38.35 (±12.27)	Baseline: *n* = 29 Post-test *N* = 27 %F = 44.4%	SITBRQ [4], OSPAQ [1]	✓	✓	Not reported	✓
Pierce et al. (2019)	Energy Company	New Zealand	39.8 [10] (28 to 58)	Baseline: *N* = 12 58.3% F	Pedometer, physical activity diary (PADs), self-report questionnaire	Not reported	✓	Not reported	✓
Puig-Ribera et al. (2015)	University	Spain	Not reported	Baseline: *N* = 129 % F not reported Ramping phase: *n* = 112 Maintenance phase: *n* = 91 Follow up phase: *n* =88	Paper diary log recording occupational sitting time	Not reported	✓	Not reported	Not reported
Rollo and Prapavessis (2020)	Large businesses, office spaces, and academic institutions	Canada	46.59 (±11.13)	Baseline: *N* = 29 93.1% F	OSPAQ [1], SBQ [5], SIT-Q 7d	✓	✓	Not reported	✓
Tobin et al. (2016)	Non-government organization and University	Australia	34.8 (±10.5)	Baseline: *N* = 26 *N* = 18 at 5 wks 89% F (16)	ActivPAL	Not reported	✓	✓	✓
Weatherson et al. (2020)	University	Canada	40.96 (±10.82)	Baseline: *N* = 24 95.8% F *N* = 20 at 3 mths *N* = 17 at 6 mths % F not reported	ActivPAL3	Not reported	✓	Not reported	✓

#### Sedentary behavior outcomes

In terms of sedentary behavior outcomes, seven studies used only a self-report outcome measure, for example, questionnaire (Coffeng et al., 2014; Graves et al., 2015; Puig-Ribera et al., 2015; Blake et al., 2019; Lithopoulos et al., 2020; Rollo and Prapavessis, 2020; Patel et al., 2021), four used a device-based measure, for example, an ActivPAL (Neuhaus et al., 2014; Tobin et al., 2016; Carter et al., 2020; Weatherson et al., 2020), and 11 used a combination of self-report and device-based measure (Chau et al., 2014; Dutta et al., 2014; De Cocker et al., 2016; Healy et al., 2016; Danquah et al., 2017; Li et al., 2017; Dunning et al., 2018; Edwardson et al., 2018; Maylor et al., 2018; Mantzari et al., 2019; Pierce et al., 2019).

All included studies showed a beneficial direction of effect on at least one sedentary behavior outcome (i.e., number of breaks, sitting time, posture change, standing time) compared with control condition (further detail in Supplementary File 2). Six studies showed changes in number of breaks (Coffeng et al., 2014; De Cocker et al., 2016; Mantzari et al., 2019; Carter et al., 2020; Rollo and Prapavessis, 2020; Patel et al., 2021). All studies showed changes in sitting time (Chau et al., 2014; Coffeng et al., 2014; Dutta et al., 2014; Neuhaus et al., 2014; Graves et al., 2015; Puig-Ribera et al., 2015; De Cocker et al., 2016; Healy et al., 2016; Tobin et al., 2016; Danquah et al., 2017; Li et al., 2017; Dunning et al., 2018; Edwardson et al., 2018; Maylor et al., 2018; Blake et al., 2019; Mantzari et al., 2019; Pierce et al., 2019; Carter et al., 2020; Lithopoulos et al., 2020; Rollo and Prapavessis, 2020; Weatherson et al., 2020; Patel et al., 2021). Seventeen studies showed changes for increased standing (Chau et al., 2014; Dutta et al., 2014; Neuhaus et al., 2014; Graves et al., 2015; De Cocker et al., 2016; Healy et al., 2016; Tobin et al., 2016; Danquah et al., 2017; Li et al., 2017; Edwardson et al., 2018; Maylor et al., 2018; Mantzari et al., 2019; Pierce et al., 2019; Carter et al., 2020; Rollo and Prapavessis, 2020; Weatherson et al., 2020; Patel et al., 2021).

### Risk of bias

Table 3 summarizes the risk of bias (RoB) assessment for each study. A mix of ratings was evident, and although no individual study was rated as having low RoB across all criteria, there were relatively few high-risk ratings. Only two studies had more than two (i.e., three) criteria rated as high risk (Coffeng et al., 2014; Blake et al., 2019). Eight studies were rated as unsure on three or more criteria for RoB (Dutta et al., 2014; Neuhaus et al., 2014; Puig-Ribera et al., 2015; De Cocker et al., 2016; Mantzari et al., 2019; Pierce et al., 2019; Lithopoulos et al., 2020; Patel et al., 2021). Across the studies, the criterion blinding of outcome assessment had the highest RoB, with nine studies assessed as high risk on this measure (Dutta et al., 2014; Neuhaus et al., 2014; Graves et al., 2015; Healy et al., 2016; Tobin et al., 2016; Danquah et al., 2017; Li et al., 2017; Blake et al., 2019; Carter et al., 2020). The criteria with the highest number of low RoB assessments were validity of outcome assessment (*n* = 16) and sequence generation (*n* = 15).

**Table 3:** Risk of bias summary by study.

**References**	**Sequence**	**Allocation**	**Blinding of**	**Incomplete**	**Selective**	**Validity of**	**Baseline**
	**generation**	**concealment**	**outcome assessment**	**outcome data**	**reporting**	**outcome reporting**	**comparability**
Blake et al. (2019)	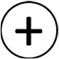	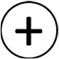	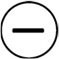	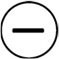	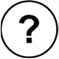	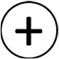	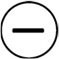
Carter et al. (2020)	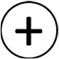	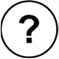	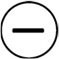	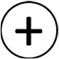	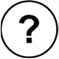	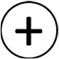	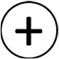
Chau et al. (2014)	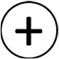	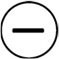	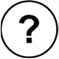	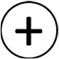	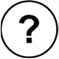	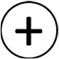	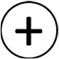
Chau et al. (2014)	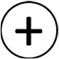	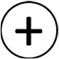	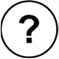	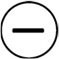	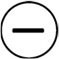	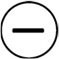	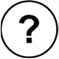
De Cocker et al. (2016)	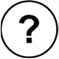	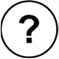	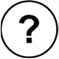	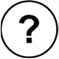	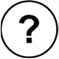	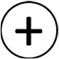	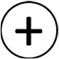
Danquah et al. (2017)	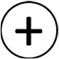	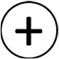	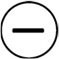	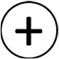	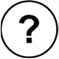	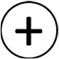	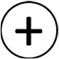
Dunning et al. (2018)	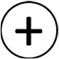	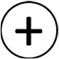	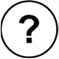	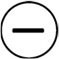	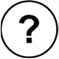	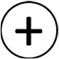	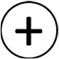
Dutta et al. (2014)	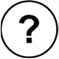	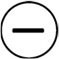	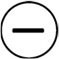	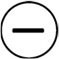	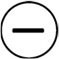	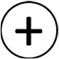	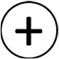
Edwardson et al. (2018)	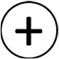	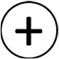	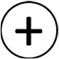	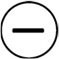	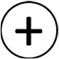	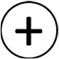	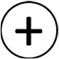
Graves et al. (2015)	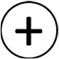	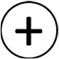	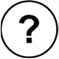	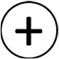	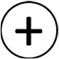	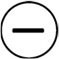	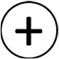
Healy et al. (2016)	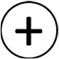	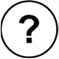	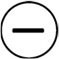	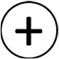	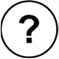	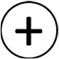	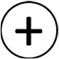
Li et al. (2017)	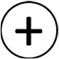	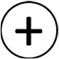	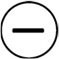	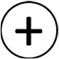	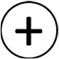	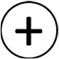	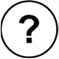
Lithopoulos et al. (2020)	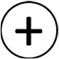	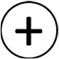	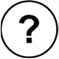	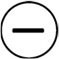	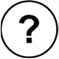	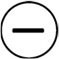	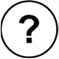
Mantzari et al. (2019)	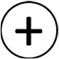	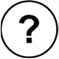	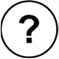	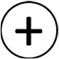	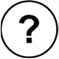	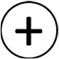	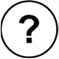
Maylor et al. (2018)	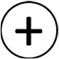	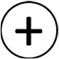	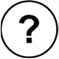	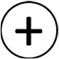	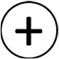	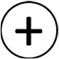	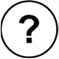
Neuhaus et al. (2014)	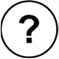	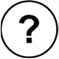	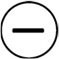	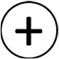	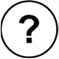	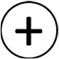	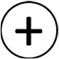
Patel et al. (2021)	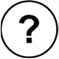	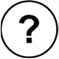	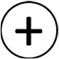	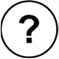	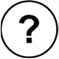	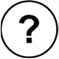	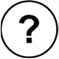
Pierce et al. (2019)	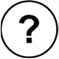	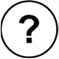	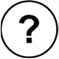	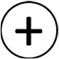	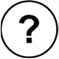	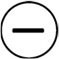	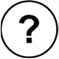
Puig-Ribera et al. (2015)	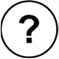	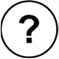	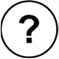	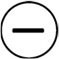	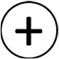	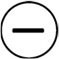	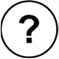
Rollo and Prapavessis (2020)	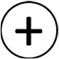	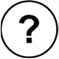	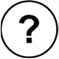	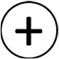	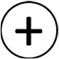	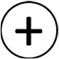	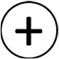
Tobin et al. (2016)	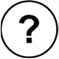	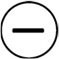	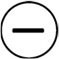	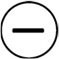	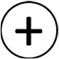	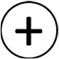	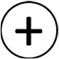
Lithopoulos et al. (2020)	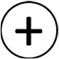	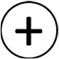	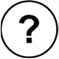	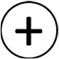	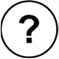	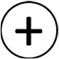	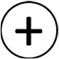

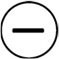
 High risk of bias; 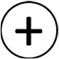 Low risk of bias; 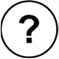 Unclear risk of bias.

### Intervention functions and BCTs evident in interventions demonstrating a beneficial direction of effect

For each intervention arm with a beneficial direction of effect we mapped the intervention content to the relevant BCW intervention function (Michie et al., 2011) and identified the included BCTs (Michie et al., 2013). Table 4 presents a synthesis of the BCW intervention functions and BCTs evident in the included studies (see Supplementary File 3 for individual studies). Of the nine BCW intervention functions, restrictions and coercion were not evident.

**Table 4:** The BCW intervention functions and BCTs evident in the included studies.

**BCW Intervention Function and definition**	**BCT and studies**	**Number of studies including BCTs**
Education (Increasing knowledge or	2.2 Feedback on behavior (Neuhaus et al., 2014; De Cocker et al., 2016; Maylor et al., 2018)	3
understanding)	5.1 Information about health consequences (Chau et al., 2014; Neuhaus et al., 2014; Puig-Ribera et al., 2015; De Cocker et al., 2016; Healy et al., 2016; Danquah et al., 2017; Edwardson et al., 2018; Maylor et al., 2018; Blake et al., 2019; Mantzari et al., 2019; Carter et al., 2020; Lithopoulos et al., 2020; Rollo and Prapavessis, 2020; Weatherson et al., 2020; Patel et al., 2021)	15
	7.1 Prompts/cues (Neuhaus et al., 2014; Danquah et al., 2017; Maylor et al., 2018; Carter et al., 2020; Rollo and Prapavessis, 2020)	5
Persuasion	5.1 Information about health consequences (Blake et al., 2019; Patel et al., 2021)	2
(Using communication to induce positive or negative feelings or stimulate action)	9.1 Credible source (Healy et al., 2016; Edwardson et al., 2018; Blake et al., 2019)	3
Incentivization (Creating an expectation of reward)	10.3 Non-specific reward (Coffeng et al., 2014)	1
Training (Imparting skills)	2.2 Feedback on behavior (Chau et al., 2014; Healy et al., 2016; Edwardson et al., 2018; Blake et al., 2019)	4
	2.3 Self-monitoring of behavior (Healy et al., 2016)	1
	2.7 Feedback on outcomes(s) of behavior (Healy et al., 2016)	1
	4.1 Instruction on how to perform the behavior (Chau et al., 2014; Coffeng et al., 2014; Dutta et al., 2014; Neuhaus et al., 2014; Graves et al., 2015; Puig-Ribera et al., 2015; De Cocker et al., 2016; Healy et al., 2016; Tobin et al., 2016; Danquah et al., 2017; Li et al., 2017; Dunning et al., 2018; Edwardson et al., 2018; Maylor et al., 2018; Blake et al., 2019; Mantzari et al., 2019; Pierce et al., 2019; Carter et al., 2020; Rollo and Prapavessis, 2020; Weatherson et al., 2020; Patel et al., 2021)	21
	6.1 Demonstration of the behavior (Graves et al., 2015; Healy et al., 2016; Danquah et al., 2017; Li et al., 2017; Blake et al., 2019; Mantzari et al., 2019; Patel et al., 2021)	7
	8.1 Behavioral practice/rehearsal (Patel et al., 2021)	1
	8.2 Behavior substitution (Dunning et al., 2018; Mantzari et al., 2019)	2
	8.3 Habit formation (Blake et al., 2019)	1
	8.7 Graded task (Puig-Ribera et al., 2015; Blake et al., 2019)	2
Enablement (Increasing means/reducing barriers to increase capability or opportunity)	1.1 Goal setting (behavior) (Coffeng et al., 2014; Dutta et al., 2014; Neuhaus et al., 2014; Puig-Ribera et al., 2015; De Cocker et al., 2016; Healy et al., 2016; Danquah et al., 2017; Edwardson et al., 2018; Maylor et al., 2018)	9
	1.2 Problem solving (Neuhaus et al., 2014; Puig-Ribera et al., 2015; De Cocker et al., 2016; Healy et al., 2016; Edwardson et al., 2018; Maylor et al., 2018; Rollo and Prapavessis, 2020; Patel et al., 2021)	8
	1.4 Action planning (De Cocker et al., 2016; Healy et al., 2016; Li et al., 2017; Edwardson et al., 2018; Rollo and Prapavessis, 2020)	5
	1.5 Review behavior goal(s) (Coffeng et al., 2014; Neuhaus et al., 2014; Puig-Ribera et al., 2015; Healy et al., 2016; Edwardson et al., 2018; Maylor et al., 2018)	6
	2.3 Self-monitoring of behavior (Puig-Ribera et al., 2015; Edwardson et al., 2018; Patel et al., 2021)	3
	3.1 Social support (unspecified) (Coffeng et al., 2014; Neuhaus et al., 2014; Puig-Ribera et al., 2015; De Cocker et al., 2016; Healy et al., 2016; Danquah et al., 2017; Li et al., 2017; Edwardson et al., 2018; Maylor et al., 2018; Carter et al., 2020; Rollo and Prapavessis, 2020)	11
	3.2 Social support (practical) (Blake et al., 2019)	1
	8.2 Behavior substitution (Edwardson et al., 2018; Patel et al., 2021)	2
	8.3 Habit formation (Li et al., 2017)	1
	8.7 Graded tasks (Puig-Ribera et al., 2015)	1
	12.1 Restructuring the physical environment (Chau et al., 2014; Healy et al., 2016; Li et al., 2017; Carter et al., 2020)	4
	12.5 Adding objects to the environment (Chau et al., 2014; Coffeng et al., 2014; Puig-Ribera et al., 2015; Healy et al., 2016; Danquah et al., 2017; Li et al., 2017; Maylor et al., 2018; Carter et al., 2020; Rollo and Prapavessis, 2020)	9
Modeling (Providing an example for people to aspire to or imitate)	6.1 Demonstration of the behavior (Healy et al., 2016; Danquah et al., 2017; Blake et al., 2019; Mantzari et al., 2019; Patel et al., 2021)	5
Environmental restructuring (Changing the physical or social context)	7.1 Prompts/cues (Coffeng et al., 2014; Dutta et al., 2014; Neuhaus et al., 2014; Puig-Ribera et al., 2015; De Cocker et al., 2016; Healy et al., 2016; Danquah et al., 2017; Li et al., 2017; Dunning et al., 2018; Edwardson et al., 2018; Maylor et al., 2018; Blake et al., 2019; Rollo and Prapavessis, 2020; Patel et al., 2021)	14
	12.1 Restructuring the physical environment (Chau et al., 2014; Coffeng et al., 2014; Dutta et al., 2014; Neuhaus et al., 2014; Graves et al., 2015; Puig-Ribera et al., 2015; De Cocker et al., 2016; Healy et al., 2016; Tobin et al., 2016; Danquah et al., 2017; Li et al., 2017; Dunning et al., 2018; Maylor et al., 2018; Blake et al., 2019; Mantzari et al., 2019; Pierce et al., 2019; Carter et al., 2020; Weatherson et al., 2020; Patel et al., 2021)	19
	12.2 Restructuring the social environment (Blake et al., 2019)	1
	12.5 Adding objects to the environment (Chau et al., 2014; Coffeng et al., 2014; Dutta et al., 2014; Neuhaus et al., 2014; Graves et al., 2015; Puig-Ribera et al., 2015; Healy et al., 2016; Tobin et al., 2016; Danquah et al., 2017; Li et al., 2017; Dunning et al., 2018; Edwardson et al., 2018; Maylor et al., 2018; Blake et al., 2019; Mantzari et al., 2019; Pierce et al., 2019; Carter et al., 2020; Rollo and Prapavessis, 2020; Weatherson et al., 2020; Patel et al., 2021)	20
Restrictions (Using rules to reduce the opportunity to engage in the target behavior)	Not used	-
Coercion (Creating an expectation of punishment or cost)	Not used	-

#### Education

Education was defined as increasing knowledge or understanding (Michie et al., 2014), and 15 of the 22 included studies used education as part of the intervention strategy (Neuhaus et al., 2014; Puig-Ribera et al., 2015; De Cocker et al., 2016; Healy et al., 2016; Danquah et al., 2017; Edwardson et al., 2018; Maylor et al., 2018; Blake et al., 2019; Mantzari et al., 2019; Carter et al., 2020; Lithopoulos et al., 2020; Rollo and Prapavessis, 2020; Weatherson et al., 2020; Patel et al., 2021). The most common educational strategy was providing information about the benefits and health consequences of sedentary behavior and physical activity. Several methods were adopted to communicate these messages including bespoke websites, posters around the office, text messages, and some studies used lectures and workshops. Feedback on behavior was also used, and this comprised individual and group feedback on sitting activity delivered *via* emails and coaching sessions. Prompts and cues were an additional educational strategy evident in these interventions. We coded education strategies as reflecting the BCTs 2.2 feedback on behavior, 5.1 information about health consequences, and 7.1 prompts/cues.

#### Persuasion

Persuasion was defined as using communication to induce positive or negative feelings or stimulate action (Michie et al., 2014), and four of the 22 included studies used persuasion as part of the intervention strategy (Healy et al., 2016; Edwardson et al., 2018; Blake et al., 2019; Patel et al., 2021). This included targeted messaging highlighting health consequences (e.g., “Break in sitting, make better working”; Patel et al., 2021). Additionally, this included supportive communication from senior colleagues to encourage engagement with the intervention (e.g., allowing time for activities and encouraging managers to filter the message down through the staff body). We coded these as reflecting the BCTs 5.1 information about health consequences and 9.1 credible source.

#### Incentivization

Incentivisation was defined as creating an expectation of reward (Michie et al., 2014), and one study used incentivisation as part of the intervention strategy (Coffeng et al., 2014). This one study encouraged participants to consider self-delivered rewards for achieving target behavior (i.e., reducing sitting time during the workday). Incentivisation strategies were coded to the BCT 10.3 non-specific reward.

#### Training

Training was defined as imparting skills (Michie et al., 2014), and 21 of the 22 included studies used training as part of the intervention strategy (Chau et al., 2014; Coffeng et al., 2014; Dutta et al., 2014; Neuhaus et al., 2014; Graves et al., 2015; Puig-Ribera et al., 2015; De Cocker et al., 2016; Healy et al., 2016; Tobin et al., 2016; Danquah et al., 2017; Li et al., 2017; Dunning et al., 2018; Edwardson et al., 2018; Maylor et al., 2018; Blake et al., 2019; Mantzari et al., 2019; Pierce et al., 2019; Carter et al., 2020; Rollo and Prapavessis, 2020; Weatherson et al., 2020; Patel et al., 2021). Examples of delivery included provision of guided exercise sessions at regular intervals throughout the day, including feedback from an instructor (e.g., led by team leader/visuals/videos); provision of strategies to break up sitting, such as using the sit-stand desk, remembering to raise the sit-stand desk each morning, walking/standing meetings, workplace challenges (e.g., step count challenge); dissemination of a training manual to support team leaders to facilitate and encourage engagement with the intervention; delivery of one-to-one coaching sessions by a health coach to identify and set goals and individual behavior change strategies including training to “listen to body” and advice about changing posture regularly; and, an individual health check report with follow up meetings. Training strategies were coded to the BCTs 2.2 feedback on behavior, 2.3 self-monitoring of behavior, 2.7 feedback on outcome(s) of behavior, 4.1 instruction on how to perform the behavior, 6.1 demonstration of the behavior, 8.1 behavioral practice/rehearsal, 8.2 behavior substitution, 8.3 habit formation, 8.7 graded task.

#### Enablement

Enablement was defined as increasing means or reducing barriers to increase capability or opportunity (Michie et al., 2014), and 15 of the 22 included studies used enablement as part of the intervention strategy (Chau et al., 2014; Coffeng et al., 2014; Dutta et al., 2014; Neuhaus et al., 2014; Puig-Ribera et al., 2015; De Cocker et al., 2016; Healy et al., 2016; Danquah et al., 2017; Li et al., 2017; Edwardson et al., 2018; Maylor et al., 2018; Blake et al., 2019; Carter et al., 2020; Rollo and Prapavessis, 2020; Patel et al., 2021). Examples of delivery included goal setting strategies (group and individual) recorded using an activity tracker or personal log or activity planning sheet (incl. coping strategies); telephone calls at regular time points delivered by an appropriate professional to support goal attainment including assessment of progress toward goals, problem-solving, action planning, adjustment/progression of goals and related behavior change strategies; an e-health programme to support reduction of sedentary behavior and goal attainment (the software remotely installed onto a work computer/laptop); and, motivational interviewing comprising discussions around participant progress toward goals, problem-solving, and adjustment of goals and behavior change strategies as necessary. Enablement strategies were coded to the BCTs 1.1 goal setting (behavior), 1.2 problem solving, 1.4 action planning, 1.5 review behavior goal(s), 2.3 self-monitoring of behavior, 3.1 social support (unspecified), 3.2 social support (practical), 8.2 behavioral substitution, 8.3 habit formation, 12.1 restructuring the physical environment, and 12.5 adding objects to the environment.

#### Modeling

Modeling was defined as providing an example for people to aspire to or imitation (Michie et al., 2014), and five of the 22 included studies used modeling as part of the intervention strategy (Healy et al., 2016; Danquah et al., 2017; Blake et al., 2019; Mantzari et al., 2019; Patel et al., 2021). Examples of delivery included provision of role models (e.g., ambassadors/team leaders/managers/team champs) who provided or demonstrated examples for participants to aspire to, to enable them to achieve goals and to implement the intervention strategies into their workday. Modeling strategies were coded to the BCT 6.1 demonstration of the behavior.

#### Environmental restructuring

Environmental restructuring was defined as changing the physical or social context, and 21 of the 22 included studies used environmental restructuring as part of the intervention strategy (Chau et al., 2014; Coffeng et al., 2014; Dutta et al., 2014; Neuhaus et al., 2014; Graves et al., 2015; Puig-Ribera et al., 2015; De Cocker et al., 2016; Healy et al., 2016; Tobin et al., 2016; Danquah et al., 2017; Li et al., 2017; Dunning et al., 2018; Edwardson et al., 2018; Maylor et al., 2018; Blake et al., 2019; Mantzari et al., 2019; Pierce et al., 2019; Carter et al., 2020; Rollo and Prapavessis, 2020; Weatherson et al., 2020; Patel et al., 2021). Examples of how environmental restructuring was delivered included provision of regular prompts encouraging staff to participate/engage in movement (daily/twice a week/weekly/based on individual dosage) with reminders/suggestions to move (e.g. onscreen/text message/stickers/step challenges); goal setting mechanisms (e.g. activity log/goal setting booklet); sit-stand desk (e.g. standard/electric/desk mount) including appropriate assessment for safe usage and provision of information about how to use safely; a device (Darma cushion) used to track sitting and provide prompts to move; and specially designed zones to encourage standing and moving (e.g. coffee bar with chairs and large plant, exercise balls, room with standing table and relaxing poster, footsteps promoting stair walking). These strategies were coded to the BCTs 7.1 prompts/cues, 12.1 restructuring the physical environment, 12.2 restructuring the social environment, and 12.5 adding objects to the environment.

### Judging the transferability of effective office-based interventions to the working at home environment using the APEASE criteria

Supplementary File 3 details the researchers' APEASE (Michie et al., 2011; West and Michie, 2022) ratings for all of the BCTs identified in each study. Table 5 shows the scores for stakeholders and research team in relation to the transferability of the intervention functions used in the included studies, and associated examples of how the intervention had been delivered in practice. Scores vary across all intervention types and examples of delivery, with most being scored as possibly transferable.

**Table 5:** Summary of APEASE scoring for stakeholders and research team.

**Intervention**	**Example of how it could be delivered**	**APEASE score**						**APEASE score**					
**type**		**expert stakeholders**						**research team**					
		**A**	**P**	**E**	**A**	**S**	**E**	**A**	**P**	**E**	**A**	**S**	**E**
Education	Materials about SB and physical activity − including benefits, health consequences, how to reduce SB, facts, tips, etc. (e.g. website/poster/leaflet/text message/lecture)	+	+		+	?	?	+	+		+	+	+
	Feedback on sitting activity along with a suggestion to break up sitting (email/coaching session)	?	+		?	?	+/?	?	?		?	+	+
Persuasion	Support for the intervention from senior management − encouraging staff involvement, allowing time for activities and encouraging managers to filter the message down through the staff body	?	?		+	?	?	?	?		+	+	+
Incentives	Self−delivered rewards for achieving target behavior	?	?		?	?	?	+	+		+	+	+
Training	Exercise sessions at regular intervals throughout the day incl. feedback from instructor (e.g led by team leader/visuals/videos)	?	?		?	?	?	?	?		?	?	+
	Strategies to break up sitting e.g. using the desk, remembering to raise the desk each morning, Walking/standing meetings, challenges	?	?		+	?	?	?	?		?	?	+
	Training manual to support team leaders to facilitate/deliver/encourage engagement with the intervention	?	?		?	?	+/?	+	+		+	+	+
	One−to−one coaching sessions delivered by a health coach to identify and set goals and individual−behavior change strategies incl. training to ‘listen to body' and advice about changing posture regularly	?	?		?	+	+	−	?		?	?	+
	Individual health check report with follow up meetings	?	?		?	+	+	−	?		?	?	+
Enablement	Goal setting strategies (group and individual) recorded using an activity tracker/personal log/activity planning sheet (incl. coping strategies)	?	+/?		+	+	+/?	?	?		?	?	+
	Telephone calls at regular time points delivered by an appropriate professional to support goal attainment involving assessment of progress toward goals, problem−solving, action planning, adjustment/progression of goals and related behavior change strategies.	?	−		−	?	?	−	?		?	?	+
	e−health programme to support reduction in SB, goal attainment (Software remotely installed onto work computer/laptop)	?	+		?	+	+	?	?		?	?	+
	Motivational interviewing comprising discussions around participant progress toward goals, problem−solving, and adjustment of goals and behavior change strategies as necessary	?	?		?	+	+/?	?	?		?	?	+
Modeling	Role models (e.g. ambassadors/team leaders/managers/team champs) to provide social support to achieve goals and to implement the intervention strategies	+	+		+	?	+	?	?		?	+	+
Environmental restructuring	Regular prompts encouraging staff to participate/engage in movement (daily/twice a week/weekly/based on individual dosage) with reminders/suggestions to move (e.g. onscreen/text message/stickers/step challenges)	+/?	+		+	?	+	?	?		?	?	+
	Goal setting mechanisms (e.g. activity log/goal setting booklet)	+/?	+		+	?	+/?	?	?		?	?	+
	Sit − stand desk (e.g. standard/electric/desk mount) incl. appropriate assessment for safe usage	?	?		+	?	?	−	−		?	?	+
	Darma cushion to track sitting and prompt movement	−	?/−		?	?	?	−	?		?	?	+
	Zones to encourage standing and moving (e.g. Coffee bar with chairs and large plant, Exercise balls, room with standing table and relaxing poster, Footsteps promote stair walking)	?	+/?		?	?	?	−	−		−	−	+

+, transferable; −, not transferable; ?, possibly transferable; +/?, split decision between respondents on transferable and possibly transferable; ?/−, split decision between respondents on not transferable and possibly transferable.

#### Education

For education, the stakeholders indicated that materials about sedentary behavior and physical activity were transferable, except in terms of safety and equity, which they indicated was potentially transferable. The research team scored this as transferable. For feedback on behavior, stakeholders indicated this to be possibility transferable, with the exception of practicability, which was indicated to be transferable. The research team scored as potentially transferable, with the exception of safety and equity, which was scored as transferable.

#### Persuasion

For persuasion, stakeholders felt that support for the intervention from senior management was potentially transferable, with the exception of acceptability, which they scored as transferable. The research team indicated this was a transferable strategy, with the exception of affordability and practicability, which was scored as potentially transferable.

#### Incentivization

The stakeholders indicated that provision of an incentive was potentially transferable. The research team scored this as transferable.

#### Training

For almost all examples of how training could be delivered, stakeholders indicated these to be potentially transferable, except for strategies to break up sitting which they scored as transferable in terms of acceptability. For the training manual, there was split opinion on equity, and one-to-one coaching sessions and individual health checks were scored as transferable in terms of equity. The research team scored the one-to-one coaching sessions and individual health checks as not transferable in terms of affordability, but possibly transferable for the practicability, acceptability, and safety. Otherwise, the training manual was scored as transferable across all constructs, and all other examples were considered to be potentially transferable.

#### Enablement

The scores for enablement were the most varied for the stakeholder group. For goal setting strategies, stakeholders were completely divided between transferable and potentially transferable in terms of practicability, side effects, and equity. But agreed this was transferable from an acceptability perspective. Regular telephone calls to support engagement with strategies to break up sitting were considered to be potentially transferable for the most part, however in terms of practicability and acceptability, this type of strategy was considered not transferable to the work at home environment. E-health programmes were considered transferable in terms of practicability, side-effects, and equity, but considered only potentially transferable in terms of affordability and acceptability. Motivational interviewing was mostly considered to be potentially transferable, however stakeholders were split on equity. For all constructs except telephone calls, the research team considered enablement strategies to be potentially transferable, with the exception of equity, which was considered to be transferable.

#### Modeling

For modeling, one example of delivery was through role models, e.g., ambassadors/team leaders/managers demonstrating the behavior and providing support and encouragement to engage with the intervention. The stakeholder group felt this was a transferable strategy, except for side effects. The research team felt this was a potentially transferable strategy except for side effects and equity, which were scored as transferable.

#### Environmental restructuring

Overall, examples of environmental restructuring were shown to have the most transferable scores by stakeholders. Prompts were considered to be transferable, except in terms of affordability, which was split between transferable and potentially transferable, and side effects, which was scored as potentially transferable. Goal setting was scored the same except for side effects, which was split between transferable and potentially transferable. Sit-stand desks were considered potentially transferable, except in terms of acceptability which was considered to be transferable. The Darma cushion was considered not transferable in terms of affordability, experts were split between not transferable and potentially transferable for practicability. Otherwise, this was considered potentially transferable. Zones to encourage standing and moving were considered potentially transferable, except for practicability that was split between transferable and potentially transferable. Scoring for the research team was more toward the potentially transferable category, however, researchers indicated sit-stand desks and the Darma cushion to be not transferable in terms of affordability. Desks were also not transferable in the context of practicability. Setting up zones for movement were not considered to be transferable at all, except for equity.

## Discussion

Working at home for at least part of the week is likely to become increasingly common for many employees. However, initial evidence suggests that working at home is likely to exacerbate already high levels of workplace sedentary behavior evident in office settings (McDowell et al., 2020; Fukushima et al., 2021). The purpose of this rapid review was to build on the growing evidence base of intervention strategies that have been effective in reducing sedentary behavior in office settings to inform intervention development to support workers in the home environment. We identified 22 high quality RCT studies, including 29 intervention arms that showed a beneficial direction of effect for at least one outcome measure of sedentary behavior in the intervention group(s) compared to the control conditions. From these studies we identified that the most common intervention functions were environmental restructuring, training, enablement and education. The most common BCTs were information on health consequences, instructions on how to perform the behavior, and restructuring the physical environment. Finally, our assessment of potential transferability of the interventions to the home working environment highlighted that educational materials, role models, incentives, and regular prompts were the most promising interventions transferable to supporting reduced sedentary behavior when working at home.

Consistent with the rapid review guidance (Garritty et al., 2021), we included only studies with a robust study design incorporating both randomization and control conditions. Further, the relatively few ratings of high risk of bias in these studies further increased our confidence in the findings. The 22 studies identified were conducted in 13 different countries reflecting the international interest in reducing workplace sedentary behavior. There was limited research in low-middle income countries, potentially limiting transferability of the findings to this context. This review included eleven studies that were also included in the 2018 Cochrane Review (Shrestha et al., 2018), but it is notable that there were eleven additional studies since the 2018 review, highlighting the growth of research in this area and that our review of promising interventions is timely.

From the 29 intervention arms, there were 1,577 participants included at baseline assessment points. The sample sizes ranged from 6 to 196, and future research should aim to recruit sufficient participants to adequately power analysis. We included studies with participants aged 18–65 years, although the average age of participants was 40 years with a relatively small variance potentially limiting the findings to this target group. This finding may suggest that this age group are most interested in reducing their sedentary behavior, and indeed, previous research has highlighted early to middle-aged adults as a high-risk group for sedentary behavior due to high workplace sedentary behavior (Strain et al., 2018). There was a range of work settings included from both public and private sectors.

In all but seven studies, sedentary behavior outcomes were assessed using device-based measurement with twelve studies also using self-report measures. Devices provide a more valid and reliable assessment of sedentary behavior (Byrom et al., 2016), and a combination of both device and self-report is recommended (Bakker et al., 2020), therefore, the prevalence of use of these measurement tools increases confidence in the findings. Every intervention showed a beneficial change in sitting time and, where reported, there was also evidence of beneficial changes in number of breaks and time spent standing. Collectively, these included interventions provide a robust body of evidence to consider in this rapid review of what type of intervention works in an office-based setting to enhance sedentary behavior.

The BCW (Michie et al., 2011) and BCT taxonomy (Michie et al., 2013) provided a useful framework to systematically classify the identified interventions. Environmental restructuring was one of the most commonly identified intervention functions, present in 21 of the studies included in our review. A previous review of interventions to reduce sitting, also noted the frequency and promise of environmental restructuring to reduce sitting in the workplace (Gardner et al., 2016). Further, workers have reported this as an acceptable and feasible approach (Hadgraft et al., 2018). The most frequently used BCTs were restructuring the physical environment and adding objects to the environment, which were implemented in a number of ways including sit-stand desks and adapting spaces to encourage standing and movement. However, in judging the potential transferability of these strategies to the home working environment, neither the stakeholders nor the researchers rated them as directly transferable across the APEASE criteria. The perceived lack of transferability is most likely because the home office is considerably different to the traditional office environment with limited space and resources. Additionally, in contrast to the office environment where employees typically have a similar environment, home office facilities can vary considerably. A 2020 study (Davis et al., 2020) exploring ergonomic set ups of employees working at home during COVID-19 found a range of “workstations” including, as examples, at dining tables, on the couch, in bed, at a treadmill. Nevertheless, it is notable that the stakeholders more consistently rated these strategies as being possibly transferable than the researchers, perhaps reflecting that some organizations are in a position to provide an enhanced home working environment.

The BCT prompts and cues was also used frequently within the environmental restructuring function, and included reminders to move using methods such as on-screen (computer) and text messages, and stickers. In their review, Gardner et al. (2016) reported that prompts and cues had relatively limited use within workplace settings, so these findings likely reflect a more recent growth in the use of such strategies, potentially due to increased availability of technology, such as apps (Dunn et al., 2018). Stakeholders evaluated this strategy as transferable, with researchers flagging “possibly transferable” indicating the potential of prompts and cues for supporting reduced sedentary behavior in the home working environment.

The intervention function of training was also evident in 21 of the 22 studies. The most commonly occurring BCTs were instruction on how to perform the behavior, and demonstration of the behavior and there were a wide range of strategies used to deliver this training. This finding differs from a previous review, which reported limited evidence of training in workplace studies (Gardner et al., 2016). However, these authors did report substantial evidence of the BCT instruction on how to perform a behavior, which we have classified as “training” (Michie et al., 2011) and this discrepancy may reflect different coding approaches. The stakeholders and researchers had similar ratings on the potential transferability of the training examples, although researchers saw greater potential in the use of a training manual. It is likely that training in how to change behavior will be important in supporting participants but there will be a need to carefully design this strategy so that it is adaptable to a home working environment.

The intervention function enablement was evident in 15 studies and included the BCTs social support (unspecified), goal setting (behavior), problem solving, and adding objects to the environment. These findings are consistent with the Gardner et al. (2016) review, in which enablement was the most frequently reported intervention function, and the same BCTs were present in workplace interventions highlighting the frequency of these approaches in the workplace. Gardner et al evaluated enablement and several of the BCTs as not promising strategies because they were included in more non-promising than promising interventions. However, the findings of this rapid review report the inclusion of enablement and these BCTs in interventions with a beneficial direction of effect, indicating the need to consider further their promise. Although the APEASE ratings were mixed, overall, both stakeholders and researchers scored enablement strategies to be at least potentially transferable. Notably, the stakeholders evaluated goal setting strategies and e-health programmes to be the most promising for transferring to the home working environment.

The intervention function of education was also evident in 15 studies. This finding is consistent with a previous review (Gardner et al., 2016), that also noted education as one of the most commonly used and promising intervention functions in workplaces, and further supports the importance of this approach. Within this intervention function, three BCTs were identified, the most frequently used being information about health consequences, and less frequently used were feedback on behavior, and prompts and cues. Although none of the education components were considered to be directly transferable across all of the APEASE criteria by both the stakeholders and researchers, there was generally an indication that this intervention function had potential to be transferred, especially materials about sedentary behavior. It is likely that the content of educational materials will be the same for workers in the workplace or in the home environment, however the delivery mechanism may need to be adapted. Specifically, posters and leaflets, may be useful in an office environment but would likely not be appropriate to deliver to employees while they are working at home. Alternatively, websites and text messages may be more useful.

The intervention functions that were less frequently used included persuasion, incentivisation, and modeling. This finding is consistent with Gardner et al. (2016) who reported that these functions were only evident in one study each in their 2016 review. Collectively, these findings indicate that there has been limited consideration of these intervention functions to reduce occupational sedentary behavior. Nevertheless, based on the stakeholder and researcher scoring there was indication that these types of intervention strategies could be transferable to the home working environment. For example, support from senior management to support behavior change interventions was evaluated as a form of persuasion that was potentially transferable. Similarly, a previous review also noted that employees perceived a “top-down” supportive approach from managers was important to provide permission to reduce sedentary behavior and facilitate culture change (Hadgraft et al., 2018). Future research could consider how best to support managers to support employees in their behavior change.

Incentivisation was only evident in one study where participants were encouraged to reward themselves for the target behavior, and this strategy was perceived as potentially transferable by the stakeholders and transferable by the researchers. Although there is limited evidence of use of this strategy in effective workplace sedentary behavior interventions, there has been increased interest in the role of incentives, and specifically financial incentives, in promoting physical activity, with some evidence that they may lead to sustained behavior change (Luong et al., 2020; Mitchell et al., 2020). Further research would be valuable to explore the potential of incentives for facilitating improvements in occupational sedentary behavior. Finally, modeling was evident in five studies and typically involved role models (e.g., ambassadors or team leaders) providing an example of how to engage in the behavior, and thus providing encouragement and support. The value of role models to support workplace behavior change has been previously noted (Hadgraft et al., 2018), and it is encouraging that the use of role models was considered transferable by the stakeholders, and potentially transferable by the researchers. In the home working environment role models will need to model the behavior in a different way from the office (e.g., modeling and encouraging standing and stretching during online meetings).

It is important to note that all but one study used a combination of intervention functions and associated BCTs, which is consistent with previous findings that suggested multi-component interventions are most effective in reducing workplace sedentary behavior (Chu et al., 2016). In transferring to the home working environment, it is also likely that a combination of intervention strategies will be most effective, and required in order to facilitate the stages of behavior change (Schwarzer and Hamilton, 2020).

Based on what is evident to work in the office environment, this study has made recommendations on what promising strategies could potentially support employees to reduce sedentary time when working at home. Clearly, further research is needed to build on these recommendations. Consistent with contemporary guidance on developing and evaluating complex interventions (Skivington et al., 2021), appropriate next steps would be to assess the feasibility and acceptability of the intervention, and then evaluate the effectiveness of the intervention using appropriate methods. Incorporating a process evaluation would also be important to answer questions around how an intervention is or is not effective (e.g., which intervention elements were most effective).

### Strengths and limitations

A strength and novel aspect of this study was the integration of expert stakeholder input into judging the transferability of the identified intervention strategies using a recognized defined framework. However, the level of agreement between the stakeholders and researchers was inconsistent. Future research adopting a more in-depth qualitative approach would be valuable in order to explore better the nuance of different contexts to understand what types of intervention strategies may work best. Such research would facilitate consideration of the impact of worker characteristics and type of work on potential transferability, rather than the more general evaluation undertaken in the current study. Further, it is acknowledged that the stakeholders did not include the full spectrum of workplace roles, and it is possible that stakeholders with different backgrounds and characteristics may perceive transferability differently. Nevertheless, it is a strength that these stakeholders had highly pertinent experience and expertise in promoting workplace health, and represented national level organizations.

In order to respond to the transformational changes in work patterns, we adopted a rapid review methodology, which necessarily meant that some steps were abbreviated. Finally, our last database search was in September 2021, and although it takes time to robustly identify, review and evaluate studies, further studies will have been published in that timeframe that can add value to this review.

## Conclusion

This rapid review makes an important and novel contribution to our understanding of what works to reduce occupational office-based sedentary behavior, and identifies potential strategies to support workers in the home environment as the world adapts to a new working landscape. Environmental restructuring, training, enablement and education were the most common interventions, but not all aspects will be easily transferable to the home working environment. Intervention strategies judged to be most promising for the home working environment were identified, including educational materials, role models, incentives, and regular prompts. Future intervention development research is needed to further adapt and evaluate these strategies in this new context.

## Data availability statement

The original contributions presented in the study are included in the article/Supplementary material, further inquiries can be directed to the corresponding author/s.

## Author contributions

AN, SM, CF, and RJ conceived the study. AN, SM, CF, DSi, and DSa designed the study, analyzed, and interpreted the data. AN, SM, CF, and DSi drafted and substantively revised the article. All authors have approved of the submitted version, and have agreed both to be personally accountable for the author's own contributions and to ensure that questions related to the accuracy or integrity of any part of the work, even ones in which the author was not personally involved, are appropriately investigated, resolved, and the resolution documented in the literature.

## Funding

This research was funded by a Medical Research Council Public Health Intervention Development Award (MR/W003511/1).

## Conflict of interest

The authors declare that the research was conducted in the absence of any commercial or financial relationships that could be construed as a potential conflict of interest.

## Publisher's note

All claims expressed in this article are solely those of the authors and do not necessarily represent those of their affiliated organizations, or those of the publisher, the editors and the reviewers. Any product that may be evaluated in this article, or claim that may be made by its manufacturer, is not guaranteed or endorsed by the publisher.

## Abbreviations

APEASE, Affordability, Practicability, Effectiveness, Acceptability, Side-Effects: Equity
BCT: Behavior Change Techniques
BCW: Behavior Change Wheel
COM-B, Capability, Opportunity: Motivation-Behavior
PRISMA: Preferred Reporting Items for Systematic Reviews and Meta-Analyses
RCT: Randomized Controlled Trial
ROB: Risk of Bias.
