# Supplementary material for: What works to reduce sedentary behavior in the office, and could these intervention components transfer to the home working environment?: A rapid review and transferability appraisal
Source: Front Sports Act Living. 2022 Jul 29;4:954639. doi: 10.3389/fspor.2022.954639 (PMC9372484; doi:10.3389/fspor.2022.954639)
Supplement: Supplementary file 2 [file Table_2.docx]

**Supplementary File 2: Sedentary behaviour outcome data of included studies showing a beneficial direction of effect**

| **Study** | **Beneficial direction of effect in occupational sedentary behaviour** | | | |
| --- | --- | --- | --- | --- |
|  | **Number of breaks** | **Sitting** | **Posture** | **Standing** |
| Blake | | | | |
| Control | Not reported | Change between pre and post: +10.34 h/w | Not reported | Not reported |
| Intervention | Not reported | Change between pre and post: +5.68 h/w  Difference of 4.66 h/w (p<0.01)  [Analyses were conducted on data contributed by study participants that completed the T1 (pre-intervention surveys); ITT sample of 196 INT gp and 86 CTRL gp] | Not reported | Not reported |
| Carter (Maxwell) | | | | |
| Control | Not reported | (min/8h workday)  Pre = 318.3 ± 66.8  Wk 8 = 344.7 ± 43.2 | Sit-to-stand transitions (n/8h workday)  Pre = 26 ± 8  Wk 8 = 24 ± 5 | (min/8h workday)  Pre = 108.4 ± 64.2  Wk 8 = 83.2 ± 34.7 |
| Intervention | Not reported | Pre = 345.0 min ± 37.6  Wk 8 = 333.1 ± 57.5 | Pre = 26 ± 8  Wk 8 = 25 ± 5 | Pre = 82.3± 36.0  Wk 8 = 93.5 ± 41.2 |
| Chau | | | | |
| Control | Not reported | ActivPAL  Pre-INT 2-:364 (73) mins/day  Pre-INT 1: 347 (59) | Not reported | ActivPAL  Pre-INT 2: 47 (27) mins/day  Pre-INT 1: 45 (28) |
| Intervention | Not reported | ActivPAL  Post-INT 3: 282 (80) mins/day  Pre-INT 2: 364 (73) | Not reported | ActivPAL  Post-INT 3: 128 (69) mins/day  Pre-INT 2: 47 (27)  Increased 65 min/day (ActivPAL) for Post-INT 3 minus Pre-INT 2  99 min/day (OSPAQ) for Post-INT 3 minus Pre-INT 2 |
| Coffeng | | | | |
| Control | Not reported | Baseline = 471.3 (SD 149.3)  6mths = 414.6 (SD 209.2)  12mths = 403.8 (SD 245.2) | Not reported | Not reported |
| 1. Social and physical environmental intervention | Not reported | Baseline = 477.3 (SD 166.4)  6mths = 380.6 (SD 221.6)  12mths = 378.6 (SD 221.8) | Not reported | Not reported |
| 1. Social environment intervention | Not reported | Baseline = 472.2 (SD 148.8)  6mths=428.5 (215.9)  12mths=365.8 (239.2) | Not reported | Not reported |
| 1. Physical environmental intervention | Not reported | Baseline = 500.8 (SD 170.4)  6mths=359.7 (SD 262.1)  12mths=367.2 (SD 249.6) | Not reported | Not reported |
| Danquah | | | | |
| Control | Not reported | Baseline=334 (min/8hr workday)  1mth=352  3mths=349 | Sit-to-stand transitions  Baseline=6.4  1mth=6.0  3mths=6.2 | Baseline=96 (min/8hr workday)  1mth=82  3mths=84 |
| Intervention | Not reported | Baseline=345  1mth=292  3mths=310 | Baseline 6.2  1mth 6.7  3mths 6.3  [Values reported are back transformed from natural log scale] | Baseline=82  1mth=132  3mths=113 |
| DeCocker | | | | |
| Control | ActivPAL  Baseline=3.0 (1.4)  1mth=3.2 (1.4)  3mths=3.3 (1.6) | ActivPAL Sitting at Work in % work hrs  Baseline=74.3 (15.5)  1mth=78.3 (11.1)  3mths=74.8 (13.5)  WSQ Sitting at Work min/day  Baseline = 281(65)  1mth=280 (50)  3mths=288(48) | Not reported | ActivPAL Standing at Work in % work hrs  Baseline=16.3 (9.3)  1mth=17.1 (7.9)  3mths=17.8 (9.0) |
| 1. Tailored intervention | ActivPAL  Baseline=3.8 (1.5)  1mth=3.7 (1.3)  3mths=4.3(1.6) | ActivPAL Sitting at Work in % work hrs  Baseline=66.8 (15.5)  1mth=71.7(14.0)  3mths=69.0 (13.7)  WSQ Sitting at Work min/day  338 (107)  279 (92)  259 (88) | Not reported | ActivPAL Standing at Work in % work hrs  Baseline=24.7 (13.5)  1mth=22.2 (9.0)  3mths=23.6 (11.7) |
| 1. Generic intervention | ActivPAL  Baseline 3.6 (1.3)  1mth 3.6 (1.4)  3mths 3.5 (1.3) | ActivPAL Sitting at Work in % work hrs  Baseline 69.0 (13.8)  1mth 71.2 (15.1)  3mths 68.8 (15.1)  WSQ Sitting at Work min/day  Baseline 288 (59)  1mth 279 (64)  3mths 280 (69) | Not reported | ActivPAL Standing at Work in % work hrs  Baseline 24.4 (11.3)  1mth 22.7 (15.4)  3mths 24.3 (14.4) |
| Dunning | | | | |
| Control | Not reported | ActivPAL hr / work day:  Post = 6.0 (5.5 to 6.4) | Not reported | ActivPAL Upright time hours/ work day:  Post = 1.9 (1.5 to 2.4) |
| Intervention | Not reported | ActivPAL hr / work day:  Post = 4.9 (4.4 to 5.4) | Not reported | ActivPAL Upright time hours/ work day:  Post = 2.9 (2.4 to 3.3) |
| Dutta | | | | |
| Control | Not reported | 67% -(24.4 min / hr) | Not reported |  |
| Intervention | Not reported | 46% of work-time- (19.6 min / hr)  Reduction of 21% (95% CI 18% to 25%)  OSPAQ= 40% less sitting (95% CI: 36% to 44%) during intervention vs control | Not reported | OSPAQ = increased 39% (95% CI: 35% to 43%) between intervention and control periods. |
| Edwardson (Waheed) | | | | |
| Control | Not reported | Mins/workday  Baseline = 354.1 (90.5)  3 months mean change from baseline = -6.17 (-30.24 to 17.91)  6 months mean change from baseline = −0.25 (−26.17 to 25.67)  12 months mean change from baseline = 9.22 (-17.65 to 36.09) | Not reported | Mins/workday  Baseline = 104.2 (74.0)  3 months mean change from baseline= 7.99 (−16.93 to 32.92)  6 months mean change from baseline = −3.94 (−27.15 to 19.28)  12 months mean change from baseline = −4.15 (−28.89 to 20.59) |
| Intervention | Not reported | Mins/workday  Baseline = 357.9 (86.6)  3 months mean change from baseline =−62.09 (−83.89 to −40.30)  Difference in change = -50.62 (-78.71 to -22.54)  6 months mean change from baseline = −61.50 (−86.47 to −36.53)  Difference in change = -64.40 (-97.31 to -31.50)  12 months mean change from baseline = -71.99 (-97.37 to -46.61)  Difference in change = -83.2 (-116.57 to - 49.98) | Not reported | Mins/workday  Baseline = 92.7 (53.9)  3 months mean change from baseline = 58.43 (38.18 to 78.67)  Difference in change = 48.91 (19.21 to 78.61)  6 months mean change from baseline = 68.34 (46.59 to 90.09)  Difference in change = 72.62 (44.80 to 100.44)  12 months mean change from baseline = 66.92 (46.04 to 87.80)  Difference in change = 66.00 (38.14 to 93.86) |
| Graves | | | | |
| Control | Not reported | (minutes/8-hour workday  Baseline = 387.0 (41.0)  4 weeks = 387.5 (78.0)  8 weeks = 402.2 (47.9) | Not reported | (minutes/8-hour workday)  Baseline = 42.5 (26.0)  4 weeks = 61.0 (76.2)  8 weeks = 43.7 (50.2) |
| Intervention | Not reported | Baseline = 385.9 (57.6)  4 weeks = 299.2 (93.4)  8 weeks = 322.0 (99.3)  Adjusted change to 4 weeks: −87.6 (−136.8 to −38.3)  Adjusted change to 8 weeks: −80.2 (−129.0 to −31.4) | Not reported | Baseline = 41.1 (35.0)  4 weeks = 141.1 (98.0)  8 weeks = 115.4 (111.6)  Adjusted change to 4 weeks:  82.2 (36.5 to 127.8)  Adjusted change to 8 weeks: 72.9 (21.2 to 124.6) |
| Healy | | | | |
| Control | Not reported |  | Not reported |  |
| Intervention | Not reported | Difference between intervention and control (min/8h)  -99.1 (95% CI -116.3 to – 81.8) at 3mths  -45.4 (95% CI -64.6 to -26.2) at 12mths | Not reported | Difference between intervention and control (min/8h)  95.2 (95% CI 79.8 to 110.5) at 3mths  42.8 (95% CI 25.8 to 59.8) at 12mths |
| Li | | | | |
| Control | Not reported | Baseline  ActivPAL: 362 (320, 403)  OSPAQ: 352 (297, 407)  Follow-up  ActivPAL: 384 (337, 432)  OSPAQ: 378 (318, 438) | Not reported | Baseline  ActivPAL: 106 (81, 131)  OSPAQ: 60 (18, 102)  Follow-up  ActivPAL: 121 (84, 158)  OSPAQ: 49 min / day (-1, 99) |
| 1. Group 2: 40 min sitting / 20 min standing | Not reported | Baseline  ActivPAL: 355 (271, 440)  OSPAQ: 377 (300, 454)  Follow-up  ActivPAL: 269 (211, 328) min / day  OSPAQ: 280 min / day (210, 349) | Not reported | Baseline  ActivPAL: 152 (63, 241)  OSPAQ: 38 (1, 74)  Follow-up  ActivPAL: 185 min / day (140, 230)  OSPAQ: 137 min / day (79, 195) |
| 1. Group 3: 30 min sitting / 30 min standing | Not reported | Baseline  ActivPAL: 356 (243, 469)  OSPAQ: 410 (352, 467)  Follow-up  ActivPAL: 276 (212, 340)  OSPAQ: 206 min / day (129, 284) | Not reported | Baseline  ActivPAL: 174 (61, 286)  OSPAQ: 29 (4, 54)  Follow-up  ActivPAL: 212 min / day (161, 263)  OSPAQ: 217 min / day (153, 282) |
| 1. Group 4: 20 min sitting / 40 min standing | Not reported | Baseline  ActivPAL: 410 (355, 465)  OSPAQ: 415 (395, 435)  Follow-up  229 min / day (167, 289)  OSPAQ: 213 min / day (141, 285) | Not reported | Baseline  ActivPAL: 98 (72, 124)  OSPAQ: 24 (8, 40)  Follow-up  ActivPAL: 268 min / day (223, 313)  OSPAQ: 194 min / day (135, 254) |
| Lithopolous | | | | |
| Control | Not reported | Wk 8: M = 7.01, SD = 3.22, p = .05, d = .83 | Not reported | Not reported |
| 1. Affective | Not reported | Wk 4: M=4.31, SD = 3.23;  Wk8: M=4.33, SD = 3.23 | Not reported | Not reported |
| 1. Instrumental | Not reported | Wk 4: M=6.92, SD = 3.21 | Not reported | Not reported |
| Mantzari | | | | |
| Control | Not reported | Baseline = 387.1 (31.1)  Follow up = 402.7 (23.5) | Sit-to-stand transitions  Baseline = 56.9 (27.9) to Follow-up 44.9 (18.6) | Baseline = 18.7 (5.6)  Follow-up = 32.4 (10.0) |
| Intervention | Not reported | Baseline = 379.9 (57.7)  Follow up = 301.3 (104.5) | Baseline = 65.4 (51.2) to Follow-up 145.1 (103.2) | Baseline = 17.8 (6.4)  Follow-up 33.6 (13.8) |
| Maylor | | | | |
| Control | Not reported | Baseline = 394.1 (380.1–408.1)  Change at 8wks = +0.9 (-20.6 to 22.5) | Sit-upright transitions  Baseline = 33.2 (30.8–35.6)  Change at 8wks = -1.9 (-5.7 to 1.7) | Baseline = 96.1 (85.7 to 106.6)  Change at 8wks = + 0.8 (-15.2 to 16.9) |
| Intervention | Not reported | Baseline = 395.0 (381.7–408.3)  Change at 8wks = -15.7 (-35.7 to 4.3) | Baseline = 33.1 (30.9 to 35.4)  Change at 8wks = 5.9 (2.5 to 9.3) | Baseline = 95.4 (85.5 to 105.3)  Change at 8 wks = + 15.7 (0.8 to 30.5) |
| Neuhaus | | | | |
| Control | Not reported | Not reported separately | Not reported separately | Not reported separately |
| 1. Multi-component group | Not reported | Mean difference at 3 months = -89 (-140, -38) vs. control | Sit-to-stands:  Mean difference at 3 months = RR=1.11 (0.87, 1.40) vs. control | Mean difference at 3 months = 93 (45, 141) vs. control. |
| 1. Workstations only group | Not reported | Mean difference at 3 Months = -33 (-84, 17) vs. control | Mean difference at 3 months = RR=1.15 (0.92, 1.45) vs. control | Mean difference at 3 months = 35 (-12, 81) vs. control |
| Patel | | | | |
| Control | Baseline: 3 (2–5)  Post-test: 3 (2–4) | Baseline: 85% (70–90)  Post-test: 85% (75–90) | Not reported | Baseline: 5% (5–10)  Post-test: 5% (5–10) |
| Intervention | Baseline: 2 (1–4)  Post-test: 3 (2–4) | Baseline: 65% (60–70)  Post-test: 60% (60–70) | Not reported | Baseline: 20% (10–20)  Post-test: 20% (16–20) |
| Pierce | | | | |
| Control | Not reported | Proportion of the work day  Weeks 1–2: Male: 77%, Female: 72%  Weeks 3–8: Male: 80%, Female: 77% | Not reported | Proportion of the work day  Weeks 1–2: Male: 0%, Female: 2%  Weeks 3–8: Male: <1%, Female: <1% |
| Intervention | Not reported | Weeks 1–2: Male: 59%, Female: 67%  Weeks 3–8: Males: 17%, Female: 30% | Not reported | Weeks 1–2: Male: 1%, Female: 1%  Weeks 3–8: Males: 32%, Female: 14% |
| Puig-Ribera | | | | |
| Control | Not reported | (minutes/ day)  Baseline = 404.6 (106)  Week 8 = 405.5 (110)  Week 19 = 402.8 (113)  Week 21 = 388.9 (120) | Not reported | Not reported |
| Intervention | Not reported | (minutes/ day)  Baseline = 446.4 (126)  Week 8 = 425.8 (120)  Week 19 = 422.9 (123)  Week 21 = 414.2 (129) | Not reported | Not reported |
| Rollo | | | | |
| Control | Break frequency (minutes)  Baseline: 136.71 (73.02)  Week 2: 125.32 (62.28)  Week4: 127.74 (64.21)  Week6: 127.94 (65.26)  Week 8: 98.71 (40.02)  Break duration (minutes)  Baseline: 4.00 (2.45)  Week 2: 3.81 (1.99)  Week4: 3.15 (1.44)  Week6: 3.76 (1.99)  Week 8: 3.65 (2.12) | (min/workday)  Baseline: 358.75 (78.26)  Week 2: 337.14 (106.94)  Week4: 329.69 (95.61)  Week6: 355.77 (74.21)  Week 8: 341.85 (76.40) | Not reported | (min/workday)  Baseline: 30.30 (24.21)  Week 2: 30.74 (18.28)  Week4: 33.49 (28.07)  Week6: 35.68 (25.47)  Week 8: 34.36 (23.49) |
| Intervention | Break frequency (minutes)  Baseline: 97.38 (52.01)  Week 2: 71.45 (26.24)  Week4: 68.90 (26.49)  Week6: 66.21 (29.60)  Week 8: 63.86 (29.74)  Break duration (minutes)  Baseline: 5.76 (4.20)  Week 2: 3.79 (5.59)  Week4: 2.95 (1.64)  Week6: 2.71 (1.33)  Week 8: 3.26 (2.25) | Baseline: 353.55 (80.65)  Week 2: 285.02 (102.64)  Week4: 278.21 (97.58)  Week6: 269.40 (115.83)  Week 8: 266.01 (104.01) | Not reported | Baseline: 41.52 (35.11)  Week 2: 60.23 (40.67)  Week4: 67.14 (44.14)  Week6: 65.18 (36.18)  Week 8: 74.08 (55.15) |
| Tobin | | | | |
| Control | Not reported | (minutes/8-h workday)  Baseline (both groups) = 392 minutes  Week 5 = 1.8 ± 34.7 | Sit-to-stand transitions  Baseline (both groups) = 26 (SD = 10)  Week 5 = 0.8 ± 10.5 | (minutes/8-h workday) Baseline (both groups) = 94 minutes;  Week 5 = 2.0 ± 10.5 |
| Intervention | Not reported | Baseline (both groups) = 392 minutes  Week 5 = –99.8 ± 65.7 | Baseline (both groups) = 26 (SD = 10)  Week 5 = –4.1 ± 8.9 | Baseline (both groups) = 94 minutes  Week 5 = 99.4 ± 83.9 |
| Weatherson | | | | |
| Control | Not reported | n/8h workday  Baseline = 5.69 (1.17)  3 months: = 5.70 (1.09)  6 months = 5.55 (1.19) | Not reported | n/8h workday  Baseline = 1.52 (0.94)  3 months = 1.54 (0.85)  6 months = 1.65 (0.90). |
| Intervention | Not reported | Baseline = 6.14hr/day (0.73)  3 months = 5.17 (1.58)  6 months = 5.26 (1.69) | Not reported | Baseline = 1.19hr/day (0.55)  3 months = 2.11 (1.40)  6 months = 2.00 (1.44) |
