# Supplementary material for: What works to reduce sedentary behavior in the office, and could these intervention components transfer to the home working environment?: A rapid review and transferability appraisal
Source: Front Sports Act Living. 2022 Jul 29;4:954639. doi: 10.3389/fspor.2022.954639 (PMC9372484; doi:10.3389/fspor.2022.954639)
Supplement: Supplementary file 3 [file Table_3.docx]

**Supplementary File 3**: Office-based interventions showing a beneficial direction of effect in reducing sedentary behaviour: BCW intervention functions, intervention components, delivery mechanisms, BCTs, and judgement on the transferability to work from home environment using APEASE criteria

| **STUDY** | **Blake, 2019 (Blake, Lai, Coman, Houdmont, & Griffiths, 2019)** | | | | | | | | |
| --- | --- | --- | --- | --- | --- | --- | --- | --- | --- |
| **BCW Intervention Function** | **Intervention component** | **Delivery mechanism** | **BCT(s)** | **Rating Based on Average Score** | | | | | |
|  |  |  |  | **A** | **P** | **E** | **A** | **S** | **E** |
| Restrictions | None | None | None |  |  |  |  |  |  |
| Education | Learning Qigong - educational materials are stated but not detailed - (increasing employee understanding about the benefits of physical activity for health and wellbeing – stated elsewhere) | Information on webpages | 5.1 Information about health consequences | + | + |  | + | + | + |
| Persuasion | Information from Qigong master / senior colleagues | Video | 9.1 Credible source  5.1 Information about health consequences | - | ? |  | + | + | + |
|  | Branding at the worksite. | Website, promotional video in canteen, posters | 9.1 Credible source  5.1 Information about health consequences | ? | ? |  | ? | + | + |
| Incentivisation | None | None | None |  |  |  |  |  |  |
| Coercion | None | None | None |  |  |  |  |  |  |
| Training | Learning Qigong – 6 X 2mins videos  Educational materials, the Qigong movement video, and instructors’ feedback were used to facilitate participants’ comprehension | Videos | 4.1 Instructions on how to perform the behaviour  6.1 Demonstration of the behaviour  2.2 Feedback on behaviour | - | ? |  | ? | ? | + |
|  | Co-production and train the trainer sessions.  Treatment manual for team leaders | Training session/ manual | 4.1 Instructions on how to perform the behaviour | ? | ? |  | ? | ? | + |
|  | Exercises designed to be undertaken twice per day (10 min) on every working day at set exercise break times – icon prompt (increased over time in terms of skill level. Intensity seems to remain constant throughout.) | Trainer and prompt by computer | 4.1 Instructions on how to perform the behaviour  8.3 Habit formation  8.7 Graded task | ? | ? |  | ? | ? | + |
| Enablement | Participants could either follow session led by team leader when prompted choose to ignore and complete session in own time guided by video |  | Choice/autonomy – but no specific BCT for this | ? | + |  | + | + | + |
|  | Group-based exercise. |  | 3.2 Social support (practical) | ? | ? |  | + | + | + |
|  | Team leaders available to discuss barriers to practice |  | 3.2 Social support (practical) | ? | ? |  | + | + | + |
| Modelling | Team leaders/ videos undertake Qigong | Video | 6.1 Demonstration of the behaviour | ? | ? |  | ? | ? | + |
| Environmental restructuring | Videos added to the environment  Treatment manual for team leaders | Video  Paper copy of manual | 12.5 Adding objects to the environment  12.1 Restructuring the physical environment | - | ? |  | ? | ? | + |
|  | On-screen prompts at specific times | Computer | 7.1 Prompts/cues | ? | ? |  | ? | + | + |
|  | Supporting website and branding -  Incl. promotional videos shown on a large screen in the canteen | Website/ branding/ Video on large screen | 12.1 Restructuring the physical environment | ? | ? |  | + | + | + |
|  | Company / senior management endorsement for regular breaks |  | 12.2 Restructuring the social environment | ? | ? |  | + | + | + |

| **STUDY** | **Carter, 2020 (Carter et al., 2020)** | | | | | | | | |
| --- | --- | --- | --- | --- | --- | --- | --- | --- | --- |
| **BCW Intervention Function** | **Intervention component** | **Delivery mechanism** | **BCT(s)** | **Rating Based on Average Score** | | | | | |
|  |  |  |  | **A** | **P** | **E** | **A** | **S** | **E** |
| Restrictions | None | None | None |  |  |  |  |  |  |
| Education | Information about the importance of reducing sitting time | e-booklet | 5.1 Information about health consequences | + | + |  | + | + | + |
|  | Prompt to take a break from sitting | Every 45 minutes appeared on screen as a bubble – open to engage or postpone | 7.1 Prompts / cues | ? | ? |  | ? | ? | + |
|  | Motivation to engage plus individual detail of number of breaks and activity minutes logged in previous week along with a suggestion to break up sitting with a walking break | Weekly email | 7.1 Prompts / cues | ? | ? |  | ? | ? | + |
| Persuasion | None | None | None |  |  |  |  |  |  |
| Incentivisation | None | None | None |  |  |  |  |  |  |
| Coercion | None | None | None |  |  |  |  |  |  |
| Training | Instruction on how to use the software | e-booklet | 4.1 Instruction on how to perform a behaviour | + | + |  | + | + | + |
| Enablement | e-health computer software designed to prompt employees to interrupt prolonged sitting with brief bouts of PA during working hours | Remotely installed onto computer | 12.5 Adding objects to the environment  12.1 Restructuring of the physical environment | ? | ? |  | ? | ? | + |
|  | Motivation to engage plus individual detail of number of breaks and activity minutes logged in previous week along with a suggestion to break up sitting with a walking break | Weekly email | 3.1 Social support (unspecified) | ? | ? |  | ? | ? | + |
| Modelling | None | None | None |  |  |  |  |  |  |
| Environmental restructuring | Software added to computer | e-health programme remotely installed onto computer | 12.5 Adding objects to the environment  12.1 Restructuring of the physical environment | ? | ? |  | ? | ? | + |

| **STUDY** | **Chau, 2014 (Chau et al., 2014)** | | | | | | | | |
| --- | --- | --- | --- | --- | --- | --- | --- | --- | --- |
| **BCW Intervention Function** | **Intervention component** | **Delivery mechanism** | **BCT(s)** | **Rating Based on Average Score** | | | | | |
|  |  |  |  | **A** | **P** | **E** | **A** | **S** | **E** |
| Restrictions | None | None | None |  |  |  |  |  |  |
| Education | None | None | None |  |  |  |  |  |  |
| Persuasion | None | None | None |  |  |  |  |  |  |
| Incentivisation | None | None | None |  |  |  |  |  |  |
| Coercion | None | None | None |  |  |  |  |  |  |
| Training | Guidance / training for using sit-stand desk |  | 4.1 Instructions on how to perform the behaviour | ? | ? |  | ? | ? | + |
|  | Ergonomic self-assessment |  | 2.2 Feedback on behaviour | ? | ? |  | ? | + | + |
|  | Advised to increase standing gradually to reduce fatigue and injury |  | 4.1 Instructions on how to perform the behaviour  5.1 Information about health consequences | ? | ? |  | ? | + | + |
| Enablement | Sit-stand desk for four weeks |  | 12.5 Adding objects to the environment  12.1 Restructuring the physical environment | - | ? |  | ? | ? | + |
| Modelling | None | None | None |  |  |  |  |  |  |
| Environmental restructuring | Sit-stand desk for four weeks | Sit-stand desk provided for four weeks | 12.5 Adding object to the environment  12.1 Restructuring the physical environment | - | ? |  | ? | ? | + |

| **STUDY** | **Coffeng, 2014 (Coffeng et al., 2014)** | | | | | | | | |
| --- | --- | --- | --- | --- | --- | --- | --- | --- | --- |
| **BCW Intervention Function** | **Intervention component** | **Delivery mechanism** | **BCT(s)** | **Rating Based on Average Score** | | | | | |
|  |  |  |  | **A** | **P** | **E** | **A** | **S** | **E** |
| Restrictions | None | None | None |  |  |  |  |  |  |
| Education | None | None | None |  |  |  |  |  |  |
| Persuasion | None | None | None |  |  |  |  |  |  |
| Incentivisation | Rewards for improving PA and relaxation (type of reward not specified) | Group motivational interview sessions (delivered by team leaders) | 10.3 Non-specific reward | ? | ? |  | ? | ? | + |
| Coercion | None | None | None |  |  |  |  |  |  |
| Training | Team leaders trained to deliver group motivational training | 2-day training course | 4.1 Instruction on how to perform the behaviour | ? | ? |  | ? | ? | + |
| Enablement | Group motivational interviewing – sessions lasted for 90mins for 3.5 months. Aim was to stimulate PA and relaxation with goal setting worksheet | Delivered by team leaders (after receiving 2-days of training) | 3.1 Social support (unspecified)  1.1 Goal setting (behaviour)  12.5 Adding objects to the environment  1.5 Review behaviour goal(s) | ? | ? |  | ? | ? | + |
|  | Participants could create or join PA and relaxation groups | Social media platform | 3.1 Social support (unspecified) | ? | ? |  | ? | ? | + |
| Modelling | None | None | None |  |  |  |  |  |  |
| Environmental restructuring | Goal setting worksheet to stimulate PA and relaxation | Through group motivational interview sessions (delivered by team leaders) | 12.5 Adding objects to the environment  7.1 Prompts / cues | ? | ? |  | ? | ? | + |
|  | Coffee bar with chairs and large plant | Installed into the office | 12.5 Adding objects to the environment  12.1 Restructuring the physical environment | - | - |  | - | ? | + |
|  | Poster with relaxing environment image | Installed into the office at the coffee bar | 12.5 Adding objects to the environment  12.1 Restructuring the physical environment | - | - |  | - | ? | + |
|  | Exercise balls | Added to the open office | 12.5 Adding objects to the environment  12.1 Restructuring the physical environment | - | - |  | - | ? | + |
|  | Curtains to divide desks and reduce noise | Added to the open office | 12.5 Adding objects to the environment  12.1 Restructuring the physical environment | - | - |  | - | ? | + |
|  | Room with standing table and relaxing poster | Specially designated meeting zone | 12.5 Adding objects to the environment  12.1 Restructuring the physical environment | - | - |  | - | ? | + |
|  | Tennis tables | Specially designated hall zone | 12.5 Adding objects to the environment  12.1 Restructuring the physical environment | - | - |  | - | ? | + |
|  | Lounge chairs for informal meetings | Specially designated hall zone | 12.5 Adding objects to the environment  12.1 Restructuring the physical environment | - | - |  | - | ? | + |
|  | Footsteps promote stair walking | Stickers placed on the floor | 12.5 Adding objects to the environment  12.1 Restructuring the physical environment | - | - |  | - | ? | + |

| **STUDY** | **Danquah, 2017 (Danquah et al., 2017)** | | | | | | | | |
| --- | --- | --- | --- | --- | --- | --- | --- | --- | --- |
| **BCW Intervention Function** | **Intervention component** | **Delivery mechanism** | **BCT(s)** | **Rating Based on Consensus Score** | | | | | |
|  |  |  |  | **A** | **P** | **E** | **A** | **S** | **E** |
| Restrictions | None | None | none |  |  |  |  |  |  |
| Education | Lecture to increase knowledge of SB and health | Lecture (assume face-to-face) | 5.1 Information about health consequences | ? | ? |  | ? | + | + |
|  | Information about SB (incl. content from lecture) | Leaflet | 5.1 Information about health consequences | + | ? |  | ? | + | + |
|  |  | Website | 5.1 Information about health consequences | ? | ? |  | ? | + | + |
|  | Regular prompts (optional) - varied content according to content of 4 strategies from workshop, also reiterated some of the examples | Emails (weekly) | 7.1 Prompts / cues | ? | ? |  | ? | ? | + |
|  |  | Texts (biweekly) | 7.1 Prompts / cues | ? | ? |  | ? | ? | + |
| Persuasion | None | None | None |  |  |  |  |  |  |
| Incentivisation | None | None | None |  |  |  |  |  |  |
| Coercion | None | None | None |  |  |  |  |  |  |
| Training | Strategies to use sit-stand desk (use of desk; breaking up prolonged sitting; walking meetings; setting common goals) | Workshop | 6.1 Demonstration of the behaviour  4.1 Instruction on how to perform the behaviour | ? | ? |  | ? | + | + |
|  | Provision of examples that could be used as part of the goal setting process (raising table each morning, moving paper basket from table. common goals incl. prompting each other) | Workshop | 4.1 Instruction on how to perform the behaviour | ? | ? |  | ? | + | + |
|  | Guidance for adapting the intervention to fit the local context | Workshop | 4.1 Instruction on how to perform the behaviour | ? | ? |  | ? | + | + |
|  | Support to implement the intervention at various levels across the organisation | Workshop | 4.1 Instruction on how to perform the behaviour | ? | ? |  | ? | + | + |
| Enablement | Common and individual goal setting | Goals incl. raising table each morning, moving paper basket from table. Common goals incl. prompting each other. Individual goals - written on personal notecard, common goals - on posters. | - 1. Goal setting (behaviour)   12.5 Adding objects to the environment | ? | ? |  | ? | ? | + |
|  | Provision of social support, making sure project and common goals discussed regularly | Ambassadors and senior managers (senior managers acted as role models) | 3.1 Social support (unspecified) | ? | ? |  | ? | ? | + |
| Modelling | Role models | Ambassadors and senior managers - provide social support, make sure project and common goals discussed regularly, managers acted as role models | 6.1 Demonstration of the behaviour | ? | ? |  | ? | ? | + |
| Environmental restructuring | Items added to the environment to support standing and moving more while working | High meeting tables in meeting rooms, offices and corridors, routes for walking meetings. | 12.5 Adding objects to the environment  12.1 Restructuring the physical environment | - | - |  | - | ? | + |
|  | Goal setting tools | Individual – personal notecard; group / common - posters | 12.5 Adding objects to the environment  12.1 Restructuring the physical environment  7.1 Prompts / cues | + | ? |  | ? | ? | + |

| **STUDY** | **DeCocker, 2016 (De Cocker, De Bourdeaudhuij, Cardon, & Vandelanotte, 2016)** | | | | | | | | |
| --- | --- | --- | --- | --- | --- | --- | --- | --- | --- |
| **BCW Intervention Function** | **Intervention component** | **Delivery mechanism** | **BCT(s)** | **Rating Based on Average Score** | | | | | |
|  |  |  |  | **A** | **P** | **E** | **A** | **S** | **E** |
| Restrictions | None | None | None |  |  |  |  |  |  |
| Education | Prior to intervention participants received:  - info about SB & health  - normative feedback on sitting  - suggestion to break SB every 30min (coded to training)  - feedback on PA | Web-based | 5.1 Information on health consequences  2.2 Feedback on behaviour | ? | + |  | + | + | + |
| Persuasion | None | None | None |  |  |  |  |  |  |
| Incentivisation | None | None | None |  |  |  |  |  |  |
| Coercion | None | None | None |  |  |  |  |  |  |
| Training | Suggestion to break SB every 30 min | Web-based | 4.1 Instruction on how to perform behaviour | ? | ? |  | ? | ? | + |
|  | Opt 1: Personalised, tailored advice on attitudes, self-efficacy, social support, knowledge, intentions, benefits/barriers. Individualised action plan created. Convert intentions to actions via SMART |  | 4.1 Instruction on how to perform behaviour | ? | + |  | + | + | + |
|  | Opt 2: generic info and tips on reducing/interrupting workplace sitting (no personalised info) |  | 4.1 Instruction on how to perform behaviour | ? | + |  | + | + | + |
| Enablement | Opt 1: Personalised, tailored advice on attitudes, self-efficacy, social support, knowledge, intentions, benefits/barriers (put this here as beyond education/training). Individualised action plan created. Convert intentions to actions via SMART | Web-based | 1.1 Goal setting (behaviour)  1.2 Problem solving  1.4 action planning  2.2 Feedback on behaviour  3.1 Social support | ? | ? |  | ? | ? | + |
| Modelling | None | None | None |  |  |  |  |  |  |
| Environmental restructuring | Computer package needed to deliver INT | Web-based | 12.1 Restructuring the physical environment | ? | ? |  | ? | ? | + |
|  | Prompts to move for main INT | Web-based | 7.1 Prompts/cues | ? | ? |  | ? | ? | + |

| **STUDY** | **Dunning, 2018 (Dunning, McVeigh, Goble, & Meiring, 2018)** | | | | | | | | |
| --- | --- | --- | --- | --- | --- | --- | --- | --- | --- |
| **BCW Intervention Function** | **Intervention component** | **Delivery mechanism** | **BCT(s)** | **Rating Based on Average Score** | | | | | |
|  |  |  |  | **A** | **P** | **E** | **A** | **S** | **E** |
| Restrictions | None | None | None |  |  |  |  |  |  |
| Education | None | None | None |  |  |  |  |  |  |
| Persuasion | None | None | None |  |  |  |  |  |  |
| Incentivisation | None | None | None |  |  |  |  |  |  |
| Coercion | None | None | None |  |  |  |  |  |  |
| Training | Text messages every 30 mins between 09:00-17:00 Monday to Friday instructing participants to stand and perform short task lasting 2-3 mins (e.g. fetch water, to nearest window, visit colleague) | Text message | 4.1 Instructions on how to the perform the behaviour  8.2 Behaviour substitution | ? | ? |  | ? | ? | + |
| Enablement | None | None | None |  |  |  |  |  |  |
| Modelling | None | None | None |  |  |  |  |  |  |
| Environmental restructuring | Text messages every 30 mins between 09:00-17:00 Monday to Friday instructing participants to stand and perform short task lasting 2-3 mins (e.g. fetch water, to nearest window, visit colleague) | Text message | 7.1 Prompts/ cues  12.5 Adding objects to the environment  12.1 Restructuring the physical environment | ? | ? |  | ? | ? | + |

| **STUDY** | **Dutta, 2014 (Dutta, Koepp, Stovitz, Levine, & Pereira, 2014)** | | | | | | | | |
| --- | --- | --- | --- | --- | --- | --- | --- | --- | --- |
| **BCW Intervention Function** | **Intervention component** | **Delivery mechanism** | **BCT(s)** | **Rating Based on Average Score** | | | | | |
|  |  |  |  | **A** | **P** | **E** | **A** | **S** | **E** |
| Restrictions | None | None | None |  |  |  |  |  |  |
| Education | None | None | None |  |  |  |  |  |  |
| Persuasion | None | None | None |  |  |  |  |  |  |
| Incentivisation | None | None | None |  |  |  |  |  |  |
| Coercion | None | None | None |  |  |  |  |  |  |
| Training | Ergonomic evaluation on proper standing / sitting height for the workstation | Ergonomic evaluation | 4.1 Instruction on how to perform the behaviour | ? | ? |  | ? | ? | + |
| Enablement | Aligned with desk use - goal of gradually reducing 50% of sitting over the month with standing time | Email reminder sent at the beginning of each week | 1.1 Goal setting (behaviour) | ? | ? |  | ? | ? | + |
| Modelling | None | None | None |  |  |  |  |  |  |
| Environmental restructuring | Adjustable sit-stand desk | Attachment mounted to front of existing desk plus work surface  Attachment mounted to back of existing desk plus work surface whole desk that is easily moved up and down.  All desks – came with anti-fatigue floor mat | 12.1 Restructuring the physical environment  12.5 Adding objects to the environment | - | - |  | ? | ? | + |
|  | Email sent at the beginning of each week reminding participants about their goal of replacing 50% of their sitting time at work with standing | Email | 7.1 Prompts/cues | ? | ? |  | ? | ? | + |

| **STUDY** | **Edwardson et al 2018 (Edwardson et al., 2018)** | | | | | | | | |
| --- | --- | --- | --- | --- | --- | --- | --- | --- | --- |
| **BCW Intervention Function** | **Intervention component** | **Delivery mechanism** | **BCT(s)** | **Rating Based on Average Score** | | | | | |
|  |  |  |  | **A** | **P** | **E** | **A** | **S** | **E** |
| Restrictions | None | None | None |  |  |  |  |  |  |
| Education | Initial group-based education seminar (around 30 minutes’ duration) was delivered, which covered the health  Consequences of sitting and the benefits of reducing and regularly breaking up sitting | In-person seminar (group) | 5.1 Information about health consequences | ? | ? |  | ? | + | + |
|  | Messages (from group seminar) were reinforced in a leaflet provided at the end of the seminar | Leaflet | 5.1 Information about health consequences | + | + |  | + | + | + |
|  | Every few months participants received posters, with either educational or motivational messages | Posters | Unclear what content was | + | ? |  | ? | + | + |
| Persuasion | Chief executive of the hospital trust showed support for the study and the intervention through regular e-newsletter sent to all staff, and through members of the Clinical Management Groups who were also asked to show support (ie, encourage involvement and allow time for intervention activities) and to filter message down to other management team leads | e-newsletter from chief exec via management groups | 9.1 Credible source | ? | ? |  | ? | + | + |
| Incentivisation | None | None | None |  |  |  |  |  |  |
| Coercion | None | None | None |  |  |  |  |  |  |
| Training | Brief training session on how to use the desk or platform and on the ergonomic set-up | In-person | 4.1 Instructions on how to perform the behaviour | ? | ? |  | ? | ? | + |
|  | Leaflet about how to use the desk and ergonomic set up was provided to reinforce these messages | Leaflet | 4.1 Instructions on how to perform the behaviour | + | + |  | + | + | + |
|  | Participants were given their baseline results from the activPAL device at the end of the seminar, which informed them of their sitting (total and prolonged), standing, and stepping time at work, and overall daily levels | In-person seminar | 2.2 Feedback on behaviour | ? | ? |  | ? | + | + |
| Enablement | Action plan and goal setting booklet and encouraged to set a goal around sitting less at work based on their activPAL feedback and to create an action plan for this to be achieved | This choice allowed flexibility for office set-up | - 1. Goal setting (behaviour)   1.4 Action planning  8.2 Behaviour substitution | ? | ? |  | ? | ? | + |
|  | DARMA cushion (Darma, CA, USA). To enable regularly tracking and self-monitoring of sitting time (total and prolonged) and be prompted (in the form of a vibration) to regularly break up sitting. This cushion, which can be placed on an office chair, is approximately  2.5 cm thick and uses Bluetooth to sync data with a mobile phone app to provide the participant with real time feedback. The frequency of the vibration prompt is  a user defined setting (eg, can be set up to vibrate every 30 or 45 minutes) | Darma cushion | 2.3 Self-monitoring of behaviour  2.2 Feedback on behaviour  7.1 Prompts/cues | - | ? |  | ? | ? | + |
|  | Every few months participants  received posters, with either educational or motivational messages. | Posters | 3.1 Social support (unspecified) | + | ? |  | ? | + | + |
|  | To provide ongoing support to participants, a trained member of the research team offered brief (about 15 minutes) coaching sessions, at month 1 and every three months thereafter to discuss progress, review goals and action plans, and discuss personal or social and group barriers | Either face-to face or by telephone at month 1 and every three months thereafter | 3.1 Social support (unspecified)  1.5 Review behaviour goals  1.4 Action planning  1.2 Problem solving | - | ? |  | ? | ? | + |
|  | After each visit for follow up measurements, participants were provided with their results from the ActivPAL device, and these were compared with the baseline data. This allowed the participants to review their progress and goals | Unclear | 2.2 Feedback on behaviour  1.5 Review behaviour goals | - | ? |  | ? | ? | + |
| Modelling | None | None | None |  |  |  |  |  |  |
| Environmental restructuring | Participants were provided with  a height adjustable desk or desk platform to enable them to sit or stand to work -  full sized electric desk (twin leg single step  stand desk 1200×800, MACOI, Kimbolton, UK), or a choice of two sizes of desk platform which sits on the existing desk | Desk | 12.5 Add objects to the environment | - | ? |  | ? | ? | + |
|  | DARMA cushion (Darma, CA, USA). To enable them to more regularly track and self-monitor their sitting time (total and prolonged) and be prompted (in the form of a vibration) to regularly break up sitting. This cushion, which can be placed on an office chair, is approximately  2.5 cm thick and uses Bluetooth to sync data with a mobile phone app to provide the participant with real time feedback. The frequency of the vibration prompt is a user defined setting (eg, can be set up to vibrate every 30 or 45 minutes) | Darma cushion | 12.5 Add objects to the environment  7.1 Prompts/cue | - | ? |  | ? | ? | + |
|  | Every few months participants received posters, with either educational or motivational messages | Posters | 12.5 Add objects to the environment  7.1 Prompts/cue | + | ? |  | ? | + | + |

| **STUDY** | **Graves, 2015 (Graves, Murphy, Shepherd, Cabot, & Hopkins, 2015)** | | | | | | | | |
| --- | --- | --- | --- | --- | --- | --- | --- | --- | --- |
| **BCW Intervention Function** | **Intervention component** | **Delivery mechanism** | **BCT(s)** | **Rating Based on Average Score** | | | | | |
|  |  |  |  | **A** | **P** | **E** | **A** | **S** | **E** |
| Restrictions | None | None | None |  |  |  |  |  |  |
| Education | None | None | None |  |  |  |  |  |  |
| Persuasion | None | None | None |  |  |  |  |  |  |
| Incentivisation | None | None | None |  |  |  |  |  |  |
| Coercion | None | None | None |  |  |  |  |  |  |
| Training | Basic face-to-face training on how to use the workstation and ergonomic information on correct workstation use | Desk supplier (Ergotron) | 6.1 Demonstration of the behaviour  4.1 Instruction on how to perform the behaviour | ? | ? |  | ? | ? | + |
|  | Ergonomic guidelines | Web link to manufacturer website (provided by research team) | 4.1 Instruction on how to perform the behaviour | + | + |  | + | + | + |
| Enablement | None | None | None |  |  |  |  |  |  |
| Modelling | None | None | None |  |  |  |  |  |  |
| Environmental restructuring | Sit-stand workstation | Installed by manufacturer | 12.1 Restructuring the physical environment  12.5 Adding objects to the environment | - | ? |  | ? | ? | + |

| **STUDY** | **Healy, 2016 (Healy et al., 2016)** | | | | | | | | |
| --- | --- | --- | --- | --- | --- | --- | --- | --- | --- |
| **BCW Intervention Function** | **Intervention component** | **Delivery mechanism** | **BCT(s)** | **Rating Based on Average Score** | | | | | |
|  |  |  |  | **A** | **P** | **E** | **A** | **S** | **E** |
| Restrictions | None | None | None |  |  |  |  |  |  |
| Education | Personalised summary of brainstorming session (organisational level strategies) | Personal email sent to participants | 5.1 Information about health consequences | ? | ? |  | ? | ? | + |
|  | Information about study aims and brainstorming of organisational level strategies and Discussion about organisational level strategies to define those most suitable for each site | Organisational level workshop with reps from each intervention site delivered to managers, team champions, OHS reps, general staff | 5.1 Information about health consequences | ? | ? |  | ? | ? | + |
| Persuasion | Six emails (sent at 2, 4, 6, 8, 10 and 12 weeks) to promote organisational level strategies (content tailored by team champ) | Email from team champ (typically worksite team leader) | 9.1 Credible source | ? | ? |  | ? | ? | + |
| Incentivisation | None | None | None |  |  |  |  |  |  |
| Coercion | None | None | None |  |  |  |  |  |  |
| Training | Coaching sessions - used to explain 'stand up, sit less, move more' intervention targets to provide feedback about the extent to which participants were meeting targets, according to their baseline assessment results, and to identify specific goals and individual-level behaviours change strategies relating to each of the key intervention messages | Coaching session | 6.1 Demonstration of the behaviour  4.1 Instruction on how to perform the behaviour  2.2 Feedback on the behaviour  2.7 Feedback on outcomes(s) of behaviour  2.3 Self-monitoring of behaviour | - | ? |  | ? | ? | + |
|  | Training to ‘listen to body’ and advised to regularly change posture | Coaching session | 4.1 Instruction on how to perform the behaviour | - | ? |  | ? | ? | + |
| Enablement | Individual coaching session 0-3 days after workstation installed | Face-to-face session delivered by health coaches | 3.1 Social support (unspecified) | - | ? |  | ? | ? | + |
|  | Personal tracker fitted to workstation for recording goals and strategies | Tracker fitted to workstation | 12.5 Adding objects to the environment  12.1 Restructuring the physical environment  1.1 Goal setting (behaviour)  1.4 Action planning | ? | ? |  | ? | ? | + |
|  | Four telephone call to support goal attainment involving assessment of progress toward goals, problem-solving, adjustment / progression of goals and related behaviour change strategies. T/c at 8 wks focused on sitting and activity outside of the workplace | Telephone call delivered by health coach at wks 2, 4, 8, and 12. | 3.1 Social support (unspecified)  1.1 Goal setting (behaviour)  1.4 Action planning  1.5 Review behaviour goal(s)  1.2 Problem solving | - | ? |  | ? | ? | + |
|  | Summary of session sent to participant following consultation | Personal email sent to participant | 3.1 Social support (unspecified) | ? | ? |  | ? | ? | + |
| Modelling | Team champs (role models) to promote organisational level strategies | Worksite team leader sent six emails (tailored by champ) with relevant massages for the team - at 2, 4, 6, 8, 10 and 12 weeks | 6.1 Demonstration of the behaviour  3.1 Social support (unspecified) | ? | ? |  | ? | ? | + |
| Environmental restructuring | Workstation for supporting sit-stand behaviour | Duel screen sit-stand workstation, with work surface accessory (for 12-months) and verbal and written instructions and tips for ergonomic posture for sitting and standing | 12.5 Adding objects to the environment  12.1 Restructuring the physical environment  7.1 Prompts / cues | - | ? |  | ? | ? | + |
|  | Indication of the recommended configuration tailored for each individual (i.e. appropriate desk height when standing / sitting) | Stickers | 12.5 Adding objects to the environment  12.1 Restructuring the physical environment  7.1 Prompts / cues | + | + |  | ? | + | + |

| **STUDY** | **Li, 2020 (Li et al., 2017)** | | | | | | | | |
| --- | --- | --- | --- | --- | --- | --- | --- | --- | --- |
| **BCW Intervention Function** | **Intervention component** | **Delivery mechanism** | **BCT(s)** | **Rating Based on Average Score** | | | | | |
|  |  |  |  | **A** | **P** | **E** | **A** | **S** | **E** |
| Restrictions | None | None | None |  |  |  |  |  |  |
| Education | None | None | None |  |  |  |  |  |  |
| Persuasion | None | None | None |  |  |  |  |  |  |
| Incentivisation | None | None | None |  |  |  |  |  |  |
| Coercion | None | None | None |  |  |  |  |  |  |
| Training | Instructions to vary work position | At ergonomic assessment – delivered by research team | 4.1 Instruction on how to perform the behaviour  6.2 Demonstration of the behaviour | ? | ? |  | ? | + | + |
|  | Instructions for how to use workstation | At ergonomic assessment – delivered by research team | 4.1 Instruction on how to perform the behaviour  6.2 Demonstration of the behaviour | ? | ? |  | ? | + | + |
| Enablement | Ergonomic assessment of workplace set up (plus secondary assessment when workplace fitted with sit-stand desk) | Research team | 12.1 Restructuring the physical environment | ? | ? |  | ? | + | + |
|  | Provision of ergonomic principles and protocol (dosage) | Visuals / pictograph | 4.1 Instruction on how to perform the behaviour  7.1 Prompts / cues  12.1 Restructuring the physical environment  12.5 Adding objects to the environment | + | + |  | + | + | + |
|  | Encouragement to adhere to allocated protocol (dosage) | Bi-weekly email | 1.4 Action planning  3.1 Social support (unspecified)  7.1 Prompts / cues  8.3 Habit formation | ? | + |  | ? | ? | + |
|  | Reminders to alert posture change at appropriate times | Tailored reminder on computer (VariDesk app) | 7.1 Prompts / cues  12.1 Restructuring the physical environment  12.5 Adding objects to the environment | ? | ? |  | ? | ? | + |
| Modelling | None | None | None |  |  |  |  |  |  |
| Environmental restructuring | Desk for supporting sit-stand behaviour | Sit-stand desk with instructions for how to use | 12.1 Restructuring the physical environment  12.5 Adding objects to the environment | - | ? |  | ? | ? | + |

| **STUDY** | **Lithopolous, 2020 (Lithopoulos et al., 2020)** | | | | | | | | |
| --- | --- | --- | --- | --- | --- | --- | --- | --- | --- |
| **BCW Intervention Function** | **Intervention component** | **Delivery mechanism** | **BCT(s)** | **Rating Based on Average Score** | | | | | |
|  |  |  |  | **A** | **P** | **E** | **A** | **S** | **E** |
| Restrictions | None | None | None |  |  |  |  |  |  |
| Education | Each group received 3 In-person 15min PowerPoint presentations with group activities and 5 minutes for questions.  Group 1: Affective benefits of reduced office sitting time through enjoyment of taking active breaks  Group 2: SB and Health e.g. sitting and CVD | PowerPoint Presentation given after the baseline, week 4 and week 8 measurements | 5.1 Information about health consequences | ? | ? |  | + | + | + |
| Persuasion | None | None | None |  |  |  |  |  |  |
| Incentivisation | None | None | None |  |  |  |  |  |  |
| Coercion | None | None | None |  |  |  |  |  |  |
| Training | None | None | None |  |  |  |  |  |  |
| Enablement | None | None | None |  |  |  |  |  |  |
| Modelling | None | None | None |  |  |  |  |  |  |
| Environmental restructuring | None | None | None |  |  |  |  |  |  |

| **STUDY** | **Mantzari, 2019 (Mantzari et al., 2019)** | | | | | | | | |
| --- | --- | --- | --- | --- | --- | --- | --- | --- | --- |
| **BCW Intervention Function** | **Intervention component** | **Delivery mechanism** | **BCT(s)** | **Rating Based on Average Score** | | | | | |
|  |  |  |  | **A** | **P** | **E** | **A** | **S** | **E** |
| Restrictions | None | None | None |  |  |  |  |  |  |
| Education | Information on impact on health of prolonged standing (NB control group given verbal info on this) | Leaflet | 5.1 Information about health consequences | + | + |  | + | + | + |
| Persuasion | None | None | None |  |  |  |  |  |  |
| Incentivisation | None | None | None |  |  |  |  |  |  |
| Coercion | None | None | None |  |  |  |  |  |  |
| Training | Correct ergonomic position when standing | Leaflet | 4.1 Instructions on how to perform the behaviour | + | + |  | + | + | + |
|  | How to gradually increase standing  time | Leaflet | 4.1 Instructions on how to perform the behaviour | + | + |  | + | + | + |
|  | How to break up sitting time | Leaflet | 4.1 Instructions on how to perform the behaviour  8.2 Behaviour substitution | + | + |  | + | + | + |
|  | Researcher gave a demo on how to use the desk | Demonstration | 6.1 Demonstration of the behaviour | ? | ? |  | ? | ? | + |
| Enablement | None | None | None |  |  |  |  |  |  |
| Modelling | Researcher gave a demo on how to use the desk | Demonstration | 6.1 Demonstration of the behaviour | ? | ? |  | ? | ? | + |
| Environmental restructuring | Full desks (Narbutas electric height-adjustable desk, product code: DHA165) were installed by professionals, after removal of participants' existing desks | Provision of desk (sit-stand) | 12.1 Restructuring the physical environment  12.5 Adding objects to the environment | - | ? |  | ? | ? | + |
|  | Desk mounts (Ergotron WorkFit-TL Desktop Workstation, product code: SKU: 33-406-085) were installed on top of participants' existing desks by a researcher | Provision of desk (desk top mount) | 12.1 Restructuring the physical environment  12.5 Adding objects to the environment | - | ? |  | ? | ? | + |
|  | Provision of leaflet with info | Leaflet | 12.5 Adding objects to the environment | + | + |  | + | + | + |

| **STUDY** | **Maylor, 2018 (Maylor, Edwardson, Zakrzewski-Fruer, Champion, & Bailey, 2018)** | | | | | | | | |
| --- | --- | --- | --- | --- | --- | --- | --- | --- | --- |
| **BCW Intervention Function** | **Intervention component** | **Delivery mechanism** | **BCT(s)** | **Rating Based on Average Score** | | | | | |
|  |  |  |  | **A** | **P** | **E** | **A** | **S** | **E** |
| Restrictions | None | None | None |  |  |  |  |  |  |
| Education | Organisational educational presentation - informed by scientific evidence on the dangers of excessive sitting and the benefits of interrupting sitting time. And brainstorming session to id and agree on strategies to reduce sitting in their workplace | From the project team | 5.1 Information about the health consequences | ? | ? |  | + | + | + |
|  | Leaflet briefly outlining the intervention  procedures, a facts sheet on the dangers of prolonged sitting | Leaflet (Goodie bag) | 5.1 Information about the health consequences | + | + |  | + | + | + |
|  | Instructions to download computer software and/or a phone app that prompted to get up and move at regular intervals | Computer software and/or a phone app | 7.1 Prompts / cues | ? | ? |  | ? | ? | + |
|  | Health check report and individual meetings | 20-minute face-to-face meeting with a member of the project team | 5.1 Information about the health consequences  2.2 Feedback on behaviour | - | ? |  | ? | ? | + |
| Persuasion | None | None | None |  |  |  |  |  |  |
| Incentivisation | None | None | None |  |  |  |  |  |  |
| Coercion | None | None | None |  |  |  |  |  |  |
| Training | Strategies to reduce sitting in the workplace | Summary email sent to all employees | 4.1 Instruction on how to perform the behaviour | + | + |  | + | + | + |
| Enablement | Step challenges - goal setting guidance, and took part in step challenge. daily steps entered into virtual leader board and spot prizes (shopping gift vouchers) were provided to increase motivation | Pedometer issued to individuals | 12.5 Adding objects to the environment  1.1 Goal setting (behaviour) | ? | ? |  | ? | ? | + |
|  | One-to-one telephone support (followed a semi-structured script to maintain intervention fidelity) - based on motivational interviewing and involved discussions around participant progress toward goals, problem-solving, and adjustment of goals and behaviour change strategies as necessary | 5 to 10 minutes provided  weekly from a member of the project team | 3.1 Social support (unspecified)  1.1 Goal setting (behaviour)  1.2 Problem solving  1.5 Review behaviour goal(s) | - | ? |  | ? | ? | + |
| Modelling | None | None | None |  |  |  |  |  |  |
| Environmental restructuring | Information card on ‘‘what your steps mean’’ | Goodie bag | 12.1 Restructuring the physical environment  12.5 Adding objects to the environment | + | + |  | + | + | + |
|  | Sticky notes to place around their workspace with self-selected reminders to encourage less sitting | Goodie bag | 12.1 Restructuring the physical environment  12.5 Adding objects to the environment  7.1 Prompts / cues | + | + |  | + | + | + |
|  | Prompt card to remind participants of sitting reduction strategies | Goodie bag | 12.1 Restructuring the physical environment  12.5 Adding objects to the environment  7.1 Prompts / cues | + | + |  | + | + | + |
|  | Prompts were displayed around the working environment encouraging employees to interrupt their sitting time and increase their steps | Poster | 12.1 Restructuring the physical environment  12.5 Adding objects to the environment  7.1 Prompts / cues | + | ? |  | ? | + | + |
|  | Changes to their working  environment in line with strategies identified during the brainstorming session to encourage movement away from the desk - Examples of these strategies included removal or relocation of personal bins and printers, and identification of workspaces or meeting areas to be used specifically for non-computer-based work to encourage movement away from the desk | Brainstorming session  Individuals were asked | 12.1 Restructuring the physical environment | ? | ? |  | ? | ? | + |

| **STUDY** | **Neuhaus, 2014 (Neuhaus, Healy, Dunstan, Owen, & Eakin, 2014)** | | | | | | | | |
| --- | --- | --- | --- | --- | --- | --- | --- | --- | --- |
| **BCW Intervention Function** | **Intervention component** | **Delivery mechanism** | **BCT(s)** | **Rating Based on Average Score** | | | | | |
|  |  |  |  | **A** | **P** | **E** | **A** | **S** | **E** |
| Restrictions | None | None | None |  |  |  |  |  |  |
| Education | Group level normative feedback in comparison to the average sitting time among Australian office workers | Organisational strategies | 2.2 Feedback on behaviour  5.1 Information about health consequences | ? | ? |  | ? | + | + |
|  | Normative individual feedback at baseline in comparison to the group’s sitting time | Normative individual feedback | 2.2 Feedback on the behaviour  5.1 Information about health consequences | ? | ? |  | ? | + | + |
|  | Initial 30-minute face-to-face coaching session was delivered at the worksite within 2 days following the workstation installation. This included a discussion of graphic feedback on the individual’s baseline sitting, standing, and moving time and collaborative goal setting in relation to the three program messages | Individual 30-minute face-to-face coaching session | 2.2 Feedback on the behaviour  7.1 Prompts / cues  5.1 Information about health consequences | - | ? |  | ? | ? | + |
| Persuasion | None | None | None |  |  |  |  |  |  |
| Incentivisation | None | None | None |  |  |  |  |  |  |
| Coercion | None | None | None |  |  |  |  |  |  |
| Training | Verbal (10-minute duration) and written instructions for how to use desk from the project manager on correct usage and how to alternate their working posture in line with OHS guidelines | Verbal and written instructions delivered by project manager | 4.1 Instruction on how to perform the behaviour | ? | ? |  | ? | ? | + |
|  | Organizational intervention – consultation with unit manager, an all-information session and manager emails to employees. Management consultation (30min) provided study rationale, details of participation and a discussion of approaches to stand up/sit less/move more |  | 4.1 Instruction on how to perform the behaviour | ? | ? |  | ? | + | + |
|  | Recommendations from university’s OHS advisor that regular postural changes should be implemented every 30 minutes | Instructions from OHS advisor | 4.1 Instruction on how to perform the behaviour | + | + |  | + | + | + |
| Enablement | Six fort-nightly e-mails were sent from the manager to staff. They supported program participation and included a study information booklet (provided by research staff). The remaining five e-mails encouraged staff to stand up, sit less, and move more and commented on strategies that appeared to be working well within the unit | E-mail templates were provided by research staff and tailored to the group by the manager. | 3.1 Social support (unspecified) | ? | ? |  | + | ? | + |
|  | An e-mail summarizing the agreed-upon goals was sent to each participant on the same day (of initial coaching session) | Email with agreed goals | 1.1 Goal setting (behaviour) | ? | ? |  | + | + | + |
|  | Three follow-up telephone calls (10 minutes each) were delivered at 1, 3, and 7weeks following the coaching session to assess goal achievement, problem-solve potential barriers, and reset goals as necessary | Telephone calls (10 mins) | 3.1 Social support (unspecified)  1.1 Goal setting (behaviour)  1.2 Problem solving  1.5 Review behaviour goal(s) | - | ? |  | ? | ? | + |
| Modelling | None | None | None |  |  |  |  |  |  |
| Environmental restructuring | Normative cues from co-workers standing at height-adjustable desk | Cues from co-workers (who were using standing desks) | 7.1 Prompts / cues  12.1 Restructuring the physical environment | ? | ? |  | ? | ? | + |
|  | Encouraged to stand up and take a break from long bouts of sitting by changing posture frequently (at least every 30 mins) | Prompt / messaging | 7.1 Prompts / cues  12.1 Restructuring the physical environment | ? | ? |  | ? | ? | + |
|  | Encouraged to substitute some sitting with standing or moving time, primarily by using the height-adjustable workstation, A sitting-to-standing ratio of approximately 50:50, accumulated through short bouts and regular postural transitions, was suggested | Prompt / messaging  Provision of height adjustable workstation | 7.1 Prompts / cues  12.1 Restructuring the physical environment | ? | ? |  | ? | ? | + |
|  | Environmental intervention strategy modified the personal physical office environment through the provision of fully installed height-adjustable workstations (WorkFit-S) with an attached work surface tray (www.ergotron.com) for each intervention participant | Height adjustable desk with attached work surface tray. | 12.5 Adding objects to the environment  12.1 Restructuring the physical environment | - | ? |  | ? | ? | + |
|  | Laminated self-monitoring tool. This Tracker was attached to the workstation, clearly visible to the participant and used during the coaching session and telephone calls for the participant to document and adjust specific goals and strategies used | Self-monitoring tool | 12.5 Adding objects to the environment  12.1 Restructuring the physical environment  7.1 Prompts / cues | ? | ? |  | ? | ? | + |

| **STUDY** | **Patel, 2021 (Patel, Banga, & Chandrasekaran, 2021)** | | | | | | | | |
| --- | --- | --- | --- | --- | --- | --- | --- | --- | --- |
| **BCW Intervention Function** | **Intervention component** | **Delivery mechanism** | **BCT(s)** | **Rating Based on Average Score** | | | | | |
|  |  |  |  | **A** | **P** | **E** | **A** | **S** | **E** |
| Restrictions | None | None | None |  |  |  |  |  |  |
| Education | Information about the dangers of sitting | Education manual (book) | 5.1 Information about health consequences | + | + |  | + | + | + |
|  | Benefits of reducing sitting - global recommendations of breaking sitting for 2 min every 60 min of sitting at work were advised. The physiological benefits  of breaking sitting on various physiological systems were explained | Education manual (book) | 5.1 Information about health consequences | + | + |  | + | + | + |
| Persuasion | Motivational messages (‘Stand More at Work & Walk for Life’; ‘Do you comply with your walking every day?’; ‘Stair every day, keep doctors away’; ‘Break in sitting, make better working’) were sent to each MOWE group participant’s mobile phones on Monday morning every week | Text message (SMS) | 5.1 Information about health consequences | ? | ? |  | ? | ? | + |
| Incentivisation | None | None | None |  |  |  |  |  |  |
| Coercion | None | None | None |  |  |  |  |  |  |
| Training | Simple desk-side exercises to break sitting periodically - Exercises: 12 low-intensity physical activities - with an illustrated format | Education manual (book) | 4.1 Instructions on how to perform the behaviour  6.1 Demonstration of the behaviour | + | + |  | + | + | + |
|  | Other PA activities that could reduce overall SB – standing meetings/lunchtime walks/ 30 mins walking | Education manual (book) | 4.1 Instructions on how to perform the behaviour | + | + |  | + | + | + |
|  | Motivational messages (‘Stand More at Work & Walk for Life’; ‘Do you comply with your walking every day?’; ‘Stair every day, keep doctors away’; ‘Break in sitting, make better working’) were sent to each MOWE group participant’s mobile phones on Monday morning every week | Text message (SMS) | 4.1 Instructions on how to perform the behaviour  8.1 Behavioural practice/rehearsal | ? | ? |  | ? | ? | + |
|  | Instructed to comply with the global walking recommendations of 30 min of walking 5–6 days a week | Education manual (book) | 4.1 Instructions on how to perform the behaviour | + | + |  | + | + | + |
|  | During the typical 8-h (08:00–17:00) workday, six breaks were recommended  (09:00, 10:00, 11:00, 12:00, 15:00, 16:00) | Education manual (book) | 4.1 Instructions on how to perform the behaviour | + | + |  | + | + | + |
| Enablement | The break activity log was added to this module, and the participants were instructed to complete the same for the next 4 weeks | Education manual (book) | 2.3 Self-monitoring of behaviour | + | + |  | + | + | + |
|  | Other PA activities that could reduce overall SB – standing meetings/lunchtime walks/ 30 mins walking | Education manual (book) | 8.2 Behavioural substitution | + | + |  | + | + | + |
|  | Motivational messages (‘Stand More at Work & Walk for Life’; ‘Do you comply with your walking every day?’; ‘Stair every day, keep doctors away’; ‘Break in sitting, make better working’) were sent to each MOWE group participant’s mobile phones on Monday morning every week | Text message (SMS) | 8.2 Behavioural substitution | ? | ? |  | ? | ? | + |
|  | Weekly SMS messages | Text message | 7.1 Prompts / cues | ? | ? |  | ? | ? | + |
|  | Participants provided with various strategies to reduce sitting | Text message/ Educational manual | 1.2 Problem solving | ? | ? |  | ? | ? | + |
| Modelling | Demonstration of the exercises  that should be done during the breaks | In-person (the primary investigator) | 6.1 Demonstration of the behaviour | ? | ? |  | ? | ? | + |
| Environmental restructuring | Motivational messages (‘Stand More at Work & Walk for Life’; ‘Do you comply with your walking every day?’; ‘Stair every day, keep doctors away’; ‘Break in sitting, make better working’) were sent to each MOWE group participant’s mobile phones on Monday morning every week | Text message (SMS) | 7.1 Prompts/cues  12.5 Adding objects to the environment  12.1 Restructuring the physical environment | ? | ? |  | ? | ? | + |
|  | Educational manual | Educational manual | 12.1 Restructuring the physical environment  12.5 Adding objects to the environment | + | + |  | + | + | + |

| **STUDY** | **Pierce, 2021 (Pierce, Legg, Godfrey, & Kawabata, 2019)** | | | | | | | | |
| --- | --- | --- | --- | --- | --- | --- | --- | --- | --- |
| **BCW Intervention Function** | **Intervention component** | **Delivery mechanism** | **BCT(s)** | **Rating Based on Average Score** | | | | | |
|  |  |  |  | **A** | **P** | **E** | **A** | **S** | **E** |
| Restrictions | None | None | None |  |  |  |  |  |  |
| Education | None | None | None |  |  |  |  |  |  |
| Persuasion | None | None | None |  |  |  |  |  |  |
| Incentivisation | None | None | None |  |  |  |  |  |  |
| Coercion | None | None | None |  |  |  |  |  |  |
| Training | Instruction on the adjustable height functions for desk | Verbal during installation | 4.1 Instruction of how to perform the behaviour | + | + |  | + | + | + |
|  | Workstation assessment by a physiotherapist for standing desk use by  the first author | Workstation assessment by physio | 4.1 Instruction of how to perform the behaviour | ? | ? |  | ? | ? | + |
| Enablement | None | None | None |  |  |  |  |  |  |
| Modelling | None | None | None |  |  |  |  |  |  |
| Environmental restructuring | Height adjust workstation | Desk with electronic height adjust function | 12.5 Adding objects to the environment  12.1 Restructuring the physical environment | - | ? |  | ? | ? | + |

| **STUDY** | **Puig-Ribera, 2015 (Puig-Ribera et al., 2015)** | | | | | | | | |
| --- | --- | --- | --- | --- | --- | --- | --- | --- | --- |
| **BCW Intervention Function** | **Intervention component** | **Delivery mechanism** | **BCT(s)** | **Rating Based on Average Score** | | | | | |
|  |  |  |  | **A** | **P** | **E** | **A** | **S** | **E** |
| Restrictions | None | None | None |  |  |  |  |  |  |
| Education | During weeks 7–8, workers are given information about the extra health benefits of walking faster at a comfortable pace and encouraged to raise their intensity of movement whenever possible (e.g., during active travel or lunch time walks) |  | 5.1 Information about health consequences | + | ? |  | ? | ? | + |
|  | Increasing employees´ awareness and knowledge of the health benefits of achieving 10,000 steps/day (i.e., preventing weight gain) and reducing sitting time (i.e., providing articles in the webpage published in the mass media or information from well-known scientific organisations) | Ecological support strategies | 5.1 Information about health consequences | + | ? |  | ? | ? | + |
| Persuasion | None | None | None |  |  |  |  |  |  |
| Incentivisation | None | None | None |  |  |  |  |  |  |
| Coercion | None | None | None |  |  |  |  |  |  |
| Training | Increasing employees´ self-efficacy by suggesting feasible strategies and encouraging them to generate innovative strategies that best enable them to sit less and move more | Ecological support strategies | 4.1 Instruction on how to perform the behaviour |  |  |  |  |  |  |
|  | Suggested strategies progress from incidental walking to short walks, and then longer walks |  | 8.7 Graded tasks |  |  |  |  |  |  |
| Enablement | Every two weeks employees challenged to progressively increase their movement by 1,000 to 3,000 daily steps above baseline |  | - 1. Goal setting (behaviour)   8.7 Graded tasks | + | ? |  | ? | ? | + |
|  | Strategies to achieve goals and break up occupational sitting time by integrating incidental walking into work tasks (e.g., moving rather than sitting during lectures and seminars, not sitting to take phone calls; weeks 1–2) |  | 1.1 Goal setting (behaviour) | + | ? |  | ? | ? | + |
|  | Progresses to short walks ranging from 5–10 minutes by targeting active mobility within University campuses (e.g., choosing the “longest route” to go to another Department within the campus; weeks 3–4) and then longer walks of +10 minutes by targeting active transport (e.g., walking to work whenever possible) or active lunch breaks (i.e., taking walks after lunch, alone or with colleagues, that fitted within a one-hour lunch break; weeks 5–6) | Maps are provided as examples of walks within and around the campus | 1.1 Goal setting (behaviour) | ? | - |  | ? | ? | + |
|  | Setting goals every two weeks for increasing step counts as means of reducing occupational sitting time | Ecological support strategies | 1.1 Goal setting (behaviour)  8.7 Graded task | + | ? |  | ? | ? | + |
|  | Monitoring the achievement of goals by logging daily step counts into the employee´s personal account (i.e., the resulting graphics provide individual feedback on progress) | Ecological support strategies | 1.5 Review behaviour goal(s)  12.5 Adding objects to the environment  2.3 Self-monitoring of behaviour | + | ? |  | ? | ? | + |
|  | Providing support strategies to achieve the targets and social networking for sharing experiences (i.e., using the blog to share personal strategies for sitting less, walking more and/or ways to overcome personal barriers) | Ecological support strategies | 3.1 Social support (unspecified)  12.5 Adding objects to the environment | ? | ? |  | ? | ? | + |
|  | Increasing employees´ self-efficacy by suggesting feasible strategies and *encouraging them to generate innovative strategies that best enable them to sit less and move more* |  | 1.2 Problem solving | ? | ? |  | ? | ? | + |
| Modelling | None | None | None |  |  |  |  |  |  |
| Environmental restructuring | During the maintenance period (weeks 9–19), W@WS sends automated emails encouraging workers to sustain sitting reductions and step count increases achieved in the ramping phase | These are sent weekly (weeks 9–12) and then fortnightly; no emails are sent during the last 3 weeks of the program | 12.5 Adding objects to the environment  7.1 Prompts / cues  12.1 Restructuring the physical environment | ? | ? |  | ? | ? | + |

| **STUDY** | **Rollo, 2020 (Rollo & Prapavessis, 2020)** | | | | | | | | |
| --- | --- | --- | --- | --- | --- | --- | --- | --- | --- |
| **BCW Intervention Function** | **Intervention component** | **Delivery mechanism** | **BCT(s)** | **Rating Based on Average Score** | | | | | |
|  |  |  |  | **A** | **P** | **E** | **A** | **S** | **E** |
| Restrictions | None | None | None |  |  |  |  |  |  |
| Education | Participants were introduced to SB incl. dangers | One-to-one counselling session via phone and an online presentation platform | 5.1 Information about health consequences | - | ? |  | ? | ? | + |
|  | Information booklet on SB. - The information booklet outlined SB as a health risk, benefits of reducing and breaking up SB, helpful strategies, and target behaviours | Information booklet on SB | 5.1 Information about health consequences | + | + |  | + | + | + |
|  | Daily tailored SB-related text messages with sedentary-related facts for six weeks | Text message (http://ohdontforget.com) | 5.1 Information about health consequences  7.1 Prompts / cues | ? | ? |  | ? | ? | + |
| Persuasion | None | None | None |  |  |  |  |  |  |
| Incentivisation | None | None | None |  |  |  |  |  |  |
| Coercion | None | None | None |  |  |  |  |  |  |
| Training | Also learned about dosage and increasing tolerance | through the one-to-one counselling session via phone and an online presentation platform | 4.1 Instructions on how to perform the behaviour | - | ? |  | ? | ? | + |
|  | Information booklet on SB. - The information booklet outlined SB as a health risk, benefits of reducing and breaking up SB, helpful strategies, and target behaviours | Information booklet | 4.1 Instructions on how to perform the behaviour | + | + |  | + | + | + |
|  | Daily tailored SB-related text messages with sedentary-related facts, as well as tips, challenges, and reminders to reduce their workplace sitting time for six weeks | Text messages (http://ohdontforget.com) | 4.1 Instructions on how to perform the behaviour | ? | ? |  | ? | ? | + |
|  | Participants received two challenges each week, one regarding breaking up sedentary time and one regarding reducing sedentary time – progressing in intensity and duration over time | Not specified | 4.1 Instructions on how to perform the behaviour | + | + |  | + | + | + |
| Enablement | Researcher delivered one-on-one behavioural counselling session (20-30 mins) via phone and an online presentation platform to set SB reduction strategies and coping strategies in an action plan - Participants were asked to form3–4 action plans specifying when, where, how, and for how long they would reduce and/or break up workplace sitting time over the next 6 weeks | One-to-one counselling session via phone and an online presentation platform | 1.4 Action planning  1.2 Problem solving  3.1 Social support (unspecified) | ? | ? |  | ? | ? | + |
|  | Participants were given the planning sheet with their action plan and coping strategies and told to display it somewhere prominent so they would be reminded of the strategies | Planning sheet | 7.1 Prompts/cues  12.5 Adding objects to the environment | ? | ? |  | ? | ? | + |
|  | As planning is an ongoing process, participants were also reminded via text message to revise and/or formulate new action and coping plans at the beginning of weeks 3 and 5 | Text-message (http://ohdontforget.com) | 1.4 Action planning  1.2 Problem solving  7.1 Prompts/cues | ? | ? |  | ? | ? | + |
|  | Tailored daily SB-related text messages with sedentary-related facts, as well as tips, challenges, and reminders to reduce their workplace sitting time for six weeks | Text-message (http://ohdontforget.com) | 7.1 Prompts/cues | ? | ? |  | ? | ? | + |
| Modelling | None | None | None |  |  |  |  |  |  |
| Environmental restructuring | Participants were given the planning sheet with their action plan and coping strategies and told to display it somewhere prominent so they would be reminded of the strategies | Action planning sheet intended to be displayed somewhere prominent | 7.1 Prompts/cues  12.5 Adding objects to the environment | ? | ? |  | ? | ? | + |
|  | Tailored daily SB-related text messages with sedentary-related facts, as well as tips, challenges, and reminders to reduce their workplace sitting time for six weeks | Text message (http://ohdontforget.com) | 7.1 Prompts/cues  12.5 Adding objects to the environment |  |  |  |  |  |  |

| **STUDY** | **Tobin, 2016 (Tobin, Leavy, & Jancey, 2016)** | | | | | | | | |
| --- | --- | --- | --- | --- | --- | --- | --- | --- | --- |
| **BCW Intervention Function** | **Intervention component** | **Delivery mechanism** | **BCT(s)** | **Rating Based on Average Score** | | | | | |
|  |  |  |  | **A** | **P** | **E** | **A** | **S** | **E** |
| Restrictions | None | None | None |  |  |  |  |  |  |
| Education | None | None | None |  |  |  |  |  |  |
| Persuasion | None | None | None |  |  |  |  |  |  |
| Incentivisation | None | None | None |  |  |  |  |  |  |
| Coercion | None | None | None |  |  |  |  |  |  |
| Training | Ergonomic assessment | With physiotherapist | 4.1 Instruction on how to perform a behaviour | - | ? |  | ? | + | + |
| Enablement | None | None | None |  |  |  |  |  |  |
| Modelling | None | None | None |  |  |  |  |  |  |
| Environmental restructuring | Sit-stand workstation fitted to desk |  | 12.1 Restructuring the physical environment  12.5 Adding objects to the environment | - | ? |  | ? | ? | + |

| **STUDY** | **Weatherson, 2020 (Weatherson, Wunderlich, & Faulkner, 2020)** | | | | | | | | |
| --- | --- | --- | --- | --- | --- | --- | --- | --- | --- |
| **BCW Intervention Function** | **Intervention component** | **Delivery mechanism** | **BCT(s)** | **Rating Based on Average Score** | | | | | |
|  |  |  |  | **A** | **P** | **E** | **A** | **S** | **E** |
| Restrictions | None | None | None |  |  |  |  |  |  |
| Education | Information on the health benefits of interrupting sitting time | Printed handout | 5.1 Information about health consequences | + | + |  | + | + | + |
| Persuasion | None | None | None |  |  |  |  |  |  |
| Incentivisation | None | None | None |  |  |  |  |  |  |
| Coercion | None | None | None |  |  |  |  |  |  |
| Training | Instructions on how to set up the converter | Printed handout | 4.1 Instruction on how to perform the behaviour | + | + |  | + | + | + |
|  | Instructions on how to use the converter to break up sitting every 20–30min | Printed handout | 4.1 Instruction on how to perform the behaviour | + | + |  | + | + | + |
| Enablement | None | None | None |  |  |  |  |  |  |
| Modelling | None | None | None |  |  |  |  |  |  |
| Environmental restructuring | A low-cost, cardboard, fixed height (20′′ x 20′′ x 21”) standing desk converter (CAD $20) to be set up alongside their regular desk in their workspace | Cardboard desk mount for use with normal desk | 12.1 Restructuring the physical environment  12.5 Adding objects to the environment | ? | ? |  | ? | ? | + |
